# The exosomal protein biomarkers auxiliary in diagnosis of interstitial lung disease

**DOI:** 10.1186/s12931-025-03326-2

**Published:** 2025-08-01

**Authors:** Ming Dong, Gaolei Hu, Xi Chen, Lingling Zhang, Yuxiao Zhang, Yanting Fang, Shuilin Liao, Yukai Wang, Qian Li, Peiyan Zheng, Bingpeng Guo, Tinpou Lai, Qun Luo, Huimin Huang, Qian Han, Baoqing Sun

**Affiliations:** 1 Present Address: State Key Laboratory of Respiratory Disease, National Clinical Research Center for Respiratory Disease, National Center for Respiratory Medicine, Department of Clinical Laboratory, Guangzhou Institute of Respiratory Health, The First Affiliated Hospital of Guangzhou Medical University, Guangzhou, Guangdong Province 510120 People’s Republic of China; 2 Guangdong Province, Guangzhou National Laboratory, Guangzhou, Guangdong Province 510005 People’s Republic of China; 3 Guangzhou Medical University, Guangzhou, Guangdong Province 511436 People’s Republic of China; 4 Department of Rheumatology and Immunology, Shantou Central Hospital, Shantou, Guangdong Province People’s Republic of China; 5 The First Affiliated Hospital of Chongqing Medical and Pharmaceutical College, Chongqing, 400016 China; 6 State Key Laboratory of Respiratory Disease, National Clinical Research Center for Respiratory Disease, National Center for Respiratory Medicine, Department of Respiratory and Critical Care Medicine, Guangzhou Institute of Respiratory Health, The First Affiliated Hospital of Guangzhou Medical University, Guangzhou, Guangdong Province China

**Keywords:** Interstitial lung disease, Liquid biopsy, Exosomal biomarker, Krebs von den Lungen 6

## Abstract

**Background:**

Exosome liquid biopsies might be a good supplement for early diagnosis of interstitial lung disease (ILD), especially those challenging cases such as connective tissue disease-ILD (CTD-ILD).

**Methods:**

We developed a circulating exosomal proteomic signature to identify novel biomarkers of ILDs combined with high-resolution CT (HRCT) examination and a new method that makes exosome testing clinically feasible. Blood-derived exosomes were extracted and characterized using a centrifugal microfluidic disc system (Exo-CMDS)-based chemiluminescence immunoassay before being subjected to proteomic analysis by mass spectrometry. Significantly differentially expressed proteins (DEPs) were identified and validated in > 600 clinical samples (collected at three hospitals) by comparing the ILD and disease/healthy control groups. Multivariable logistic regression (LR) analysis was implemented to test the diagnostic performance of the selected biomarkers either alone or in combination.

**Results:**

Candidate biomarkers KL-6, CAPN2, SP-B were selected from the top DEPs. An LR model that combined exosomal KL-6/CAPN2/SP-B levels performed well in both the discovery (AUC = 0.987, 95%CI = 0.975–0.998) and validation (AUC = 0.936, 95%CI = 0.911–0.960) sets. The LR model based on the three biomarkers exhibited markedly better diagnostic performance (AUC = 0.880, 95%CI = 0.834–0.925) in serum-KL-6-negative ILD, than the conventional serum-KL-6-based method and could also accurately diagnose connective tissue disease associated-ILD (CTD-ILD) in the context of CTD.

**Conclusion:**

The circulating exosomal protein detection system used in this study represents a valuable tool for identifying promising exosomal biomarkers for ILD and holds promise for improving the diagnosis and prognosis of patients with ILD in the future.

**Supplementary Information:**

The online version contains supplementary material available at 10.1186/s12931-025-03326-2.

## Introduction

Multidisciplinary discussion (MDD) in the diagnosis of interstitial lung diseases (ILD) had been reported to reclassify community-based diagnoses 40–53% of the time, suggesting that diagnosis remains challenging [1]. Symptoms of early interstitial pneumonia are uncharacteristic and overlap with those of common pulmonary complaints and non-pulmonary diseases [2]. Indeed, of the 600 patients with ILD who participated in a 2018 study, 55% had more than one misdiagnosis, and 38% had more than two misdiagnoses [3]. Specially, in some challenging cases, connective tissue disease-ILD (CTD-ILD) may represent over 55% of these reclassified cases [4].

In a previous study, 71 cases of interstitial lung abnormality (ILA) were detected in 781 lung cancer screening high-resolution computed tomography (HRCT) scans. however, only 64% of these ILA cases were reported by radiologists and < 30% of the patients received a pulmonary referral [5]. A comparative study on circulating diagnostic biomarkers for ILD identified serum Kreb von den Lungen-6 (KL-6) and matrix metalloproteinase (MMP)−7 levels as independent predictors of idiopathic pulmonary fibrosis (IPF) [6]. Moreover, serum KL-6 levels were raised in patients with CTD-ILD and positively correlated with ILD severity [7]. At present, the clinical sensitivity of KL-6 varies greatly (39%–99%), possibly due to differences in sampling and testing methods [8, 9]. Thus, effective molecular definitions extending beyond MDD-determined syndromes are needed as complement to HRCT.

Exosome-based liquid biopsies have recently emerged as a promising tool for the non-invasive diagnosis of certain lung diseases [10]. Comparing with plasma free proteins, the original protein structure might be protected to avoid being cleaved by enzymes in exosomes because of the protection via vesicular structures from phospholipid. Advancements in exosome isolation methods from clinical specimens have greatly increased the accuracy of this approach [11]. Elevated exosome numbers have been detected in the bronchoalveolar lavage fluid (BALF) of patients with pulmonary sarcoidosis, a chronic inflammatory form of ILD [12, 13]. Moreover, these BALF-derived exosomes stimulated monocytes to release immune mediators such as interleukin (IL)−1, IL-6, CC-chemokine ligand (CCL) 2, and tumor necrosis factor [14]. Thus, exosomes may contribute to lung disease by carrying cargoes that induce inflammatory responses in immune and lung epithelial cells.

Given the established association between exosomes and lung disease, we sought to find out whether exosomal proteins could be used as a reliable diagnostic method for patients with ILD, especially those challenging cases such as CTD-ILD. Based on this, we hoped to achieve an efficient, cheap, and fast detection method for these exosomal biomarkers and realize clinical application. Furthermore, the exosomal proteins involved could provide insights into pathobiological mechanisms.

## Methods

### Study design and participants

This multicenter case–control study was approved by the institutional review boards at the First Affiliated Hospital of Guangzhou Medical University (GYFYY, ES-2023–129-02, approved on 2023/08/16), Shantou Central Hospital (STC, 2022 Scientific Research No. 037, approved on 2022/7/14), and the First Affiliated Hospital of Chongqing Medical and Pharmaceutical College (CQMPC, Ethics Review 2021 No. 1, approved on 2021/01). The ethical approvals were granted under the reference numbers mentioned above and the IRB confirmed that the study complied with national ethical standards and the Declaration of Helsinki. All participants provided written informed consent prior to their inclusion in the study.

Serum samples were primarily selected from the biorepository by researchers based on medical records, sample volume, and whether the informed consent requirements were met. Diagnoses were definitively established by a multidisciplinary panel of respiratory specialists through consensus MDD, integrating guideline-directed HRCT interpretation with comprehensive clinical evaluation [15]. The discovery set consisted exclusively of patients recruited at GYFYY (*n* = 168). The validation set comprised patients from three tertiary centers: Guangzhou Medical University (GYFYY; *n* = 287), Shantou Central Hospital (STC; *n* = 113), and Chongqing Medical and Pharmaceutical College (CQMPC; *n* = 40).

The ILD group included patients with CTD-ILD, IPF, idiopathic interstitial pneumonia (IIP) other than IPF, interstitial pneumonia with autoimmune features (IPAF), or other forms of ILD.Patients classified as having CTD-ILD met the diagnostic criteria established by Kondoh et al. in 2020 [16]. Diagnosis of IPF adhered to the 2022 ATS/ERS/JRS/ALAT guidelines [17]. Other IIP were classified according to the 2013 international consensus criteria [18], while cases meeting IPAF were diagnosed using the 2015 ERS/ATS research criteria [19].

The disease control (DC) group encompassed connective tissue disorders (CTD, primarily from STC and diagnosed according to the ACR/EULAR guidelines [20–22]), chronic obstructive pulmonary pulmonary disease (COPD), lung cancer, and pulmonary infections (predominantly from GYFYY). The DC group was established based on the clinical rationale that ILD can secondarily develop in these conditions, primarily due to shared pathological mechanisms among them, such as immune dysregulation, inflammatory cascades, or tissue injury.

Healthy control (HC) samples were obtained from individuals undergoing routine health examinations at the physical examination departments of the three participating hospitals. These individuals were screened based on CT results showing no significant abnormalities and the absence of any notable respiratory distress. Fibrotic lung tissue and controls were obtained from recipients and donors in lung transplantation surgery from the Biorepository and Precision Pathology Center (GYFYY). The acquisition of these tissues was conducted under GYFYY Institutional Review Board (IRB)-approved protocol (Ethics Review 2022 No. 88, approved on 2022/07/29).

Biochemical data collected included levels of Krebs von den Lungen-6 (KL-6), carbohydrate antigen 125 (CA125), carbohydrate antigen 15–3 (CA15-3), carbohydrate antigen 199 (CA199), C-reactive protein (CRP), D-dimer, lactate dehydrogenase (LDH), triglycerides, high-density lipoprotein (HDL), low-density lipoprotein (LDL), blood glucose (Glu), and blood coagulation parameters. Only independent samples were used; repeated longitudinal samples from individual patients were excluded.

### Statistical analysis

Differential expression analyses were accomplished using the"edgeR"package in R. Categorical data were presented as frequencies and percentages, whereas continuous variables were depicted as medians with interquartile ranges (IQRs). Comparison between groups employed the Mann–Whitney U-test and Wilcoxon rank-sum test. A statistical significance threshold was set at *p* < 0.05. Power calculation was undertaken with G-Power software, version 3.1. Statistical analyses were executed utilizing IBM SPSS Statistics version 26.0 for logistic regression (LR), while GraphPad Prism version 8.0.2 facilitated ROC curve analyses and graphical illustrations.

### Extended methodology

Extended methodology is available in the Supplementary Material (file-Supplemental methods).

## Results

### Patient disposition and baseline demographics

To search for exosomal biomarkers specific to ILD, we established the ILD, DC, and HC groups and performed a MS-based proteomic analysis in a “Discovery set” and further validated the results using a “Validation set”. The clinical characteristics of these sets are shown in Table 1 and Table S1. the discovery set comprised 168 patients (45.2% male, median age 54.0 years) and the validation set comprised 440 patients (45.2% male, median age 56.0 years). About 30% of patients were former smokers at inclusion. Differences in indicators of inflammation, coagulation, and tumor, as well as blood lipid and KL-6 levels were observed between the discovery and validation sets. Meanwhile, the CA125, CA15-3, CA199, and KL-6 levels were significantly different among the three groups (Table 1). IPAF (*n* = 29) was the most common ILD in the discovery set, while CTD-ILD (*n* = 102) was the most common ILD in the validation set. Lung cancer (*n* = 22) accounted for a large proportion of cases in the discovery set, while in the validation set, the distribution of diseases in the DC group was relatively even (Table S1).

**Table 1:** Baseline characteristics for the discovery and validation set

Characteristics	Discovery Set				Validation Set			
	HC(*n* = 31)	DC (*n* = 47)	ILD (*n* = 90)	*p* value	HC (*n* = 93)	DC (*n* = 135)	ILD (*n* = 212)	*p* value
**Age-years**	48.0 (39.5–50.0)	61.0 (48.5–64.5)	56.0 (44.0–63.0)	0.0008^a^	47.0 (34.0–55.0)	57.0 (46.0–66.5)	59.0 (50.0–67.0)	< 0.0001^a^
**Male-sex**	15 (48.4%)	22 (46.8%)	39 (43.3%)	0.8596^b^	57 (61.3%)	76 (56.3%)	108 (50.9%)	0.2258^b^
**BMI-Kg/m2**	21.2 (19.3–23.5)	22.4 (19.9–25.2)	23.2 (19.9–25.1)	0.0794^a^	22.4 (19.7–23.8%)	23.5 (21.3–25.7)	22.5 (21.5–25.4)	0.0082^a^
**Ever smoker**	4 (12.9%)	19 (40.4%)	26 (28.9%)	0.0325^b^	46 (34.1%)	12 (12.9%)	74 (35.4%)	0.0002^b^
**Serum index detection**								
CRP-mg/L	0.4 (0.2–0.8)	0.4 (0.2–0.8)	0.3 (0.1–1.0)	0.8986^a^	0.5 (0.3–0.7)	0.5 (0.2–1.1)	0.7 (0.2–2.5)	0.0607^a^
D_Dimer-ug/L	291.0 (177.5–362.5)	395.0 (305.5–653.0)	361.0 (241.5–678.2)	0.0124^a^	284.0 (221.0–407.0)	378.0 (243.9–586.0)	486.5 (294.0–976.2)	< 0.0001^a^
LDH-U/L	145.0 (137.0–162.0)	179.3 (153.3–210.3)	222.7 (178.8–281.2)	< 0.0001^a^	153.0 (131.0–171.6)	163.8 (137.0–212.6)	216.0 (184.8–276.5)	< 0.0001^a^
TG-mmol/L	1.1 (0.9–1.5)	1.1 (0.9–1.4)	1.4 (1.0–1.8)	0.0655^a^	1.1 (0.8–1.7)	1.2 (0.9–1.6)	1.6 (1.2–2.2)	< 0.0001^a^
HDL-C-mmol/L	1.5 (1.2–1.6)	1.3 (1.1–1.5)	1.1 (1.0–1.4)	0.0022^a^	1.4 (1.2–1.6)	1.3 (1.1–1.5)	1.2 (1.0–1.4)	< 0.0001^a^
LDL-C-mmol/L	3.3 (2.8–3.6)	3.2 (2.5–3.5)	3.2 (2.7–3.8)	0.4606^a^	3.4 (3.0–3.8)	3.3 (2.9–3.8)	3.0 (2.6–3.4)	< 0.0001^a^
GLU-mmol/L	5.0 (4.7–5.6)	5.1 (4.6–5.6)	5.0 (4.5–5.8)	0.9521^a^	5.0 (4.7–5.3)	5.2 (4.7–5.7)	5.0 (4.5–6.3)	0.2327^a^
PT–s	12.8 (12.4–12.9)	13.1 (12.7–13.7)	13.3 (12.7–13.8)	0.0013^a^	12.7 (12.4–13.1)	13.0 (12.4–13.5)	13.2 (12.5–13.8)	0.0002^a^
APTT-s	36.0 (33.8–38.0)	35.8 (34.1–39.5)	36.9 (33.4–40.5)	0.8809^a^	36.0 (34.3–37.6)	36.8 (34.3–40.0)	36.5 (33.7–39.8)	0.2803^a^
TT-s	17.3 (16.7–17.7)	16.8 (16.3–17.9)	17.6 (16.8–18.2)	0.0392^a^	16.6 (16.2–17.1)	16.8 (16.3–17.4)	17.0 (16.2–17.8)	0.0159^a^
FIB-g/L	2.8 (2.5–3.4)	3.4 (3.0–4.0)	3.5 (2.8–4.5)	0.0014^a^	3.2 (2.9–3.6)	3.4 (2.8–4.1)	3.8 (3.2–4.7)	< 0.0001^a^
CA199-U/mL	8.4 (3.9–15.3)	12.0 (7.9–22.6)	17.8 (8.2–30.4)	0.0008^a^	8.9 (5.3–12.3)	10.7 (6.7–21.7)	17.5 (8.0–34.1)	< 0.0001^a^
CA15-3-U/mL	12.9 (9.7–18.4)	10.9 (9.1–19.5)	15.6 (11.4–47.8)	0.0007^a^	7.7 (5.8–12.6)	10.6 (7.2–17.2)	29.6 (15.9–51.6)	< 0.0001^a^
CA125-U/mL	15.4 (11.6–18.1)	15.7 (11.1–20.1)	19.2 (12.0–43.8)	0.0066^a^	9.8 (7.7–14.2)	13.2 (9.6–22.7)	22.7 (13.2–47.4)	< 0.0001^a^
KL-6-U/mL	359.0 (242.5–444.0)	313.0 (246.0–378.0)	619.0 (395.5–1530.0)	< 0.0001^a^	300.0 (259.0–393.0)	320.0 (238.0–427.5)	1013.9 (562.2–1860.0)	< 0.0001^a^

Data are median (IQR)-(%)

*APTT* Activated partial thromboplastin time, *CRP* C-reaction protein, *CA199* Carbohydrate antigen 199, *CA15-3* Carbohydrate antigen 15–3, *CA125* Carbohydrate antigen 125, *DC* Disease control, *FIB* Fibrinogen, *GLU* Glucose, *HDL-C* High-density lipoprotein cholesterol, *HC* Healthy control, *ILD* Interstitial lung disease, *KL_6* Krebs Von den Lungen-6, *LDL-C* Low-density lipoprotein cholesterol, *PT* Prothrombin time, *TG* Triglyceride, *TT* Thrombin time

^a^Kruskal-Wallis

^b^Pearson

### Screening and selection of exosomal biomarkers for ILD

**Fig. 1:**
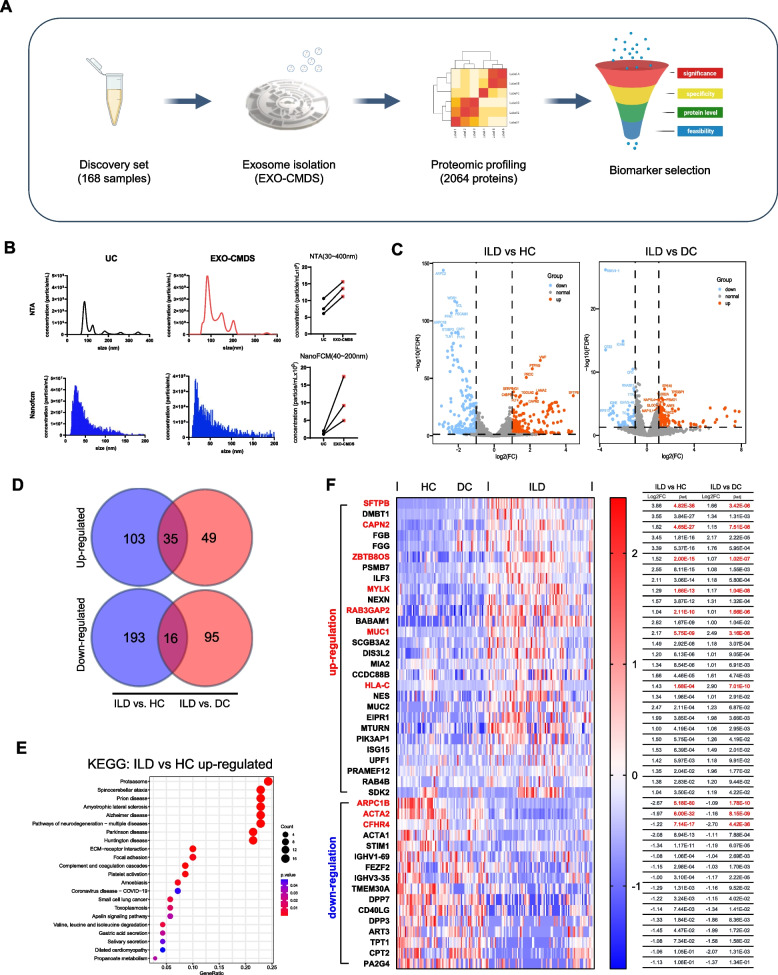
Screening and selection of exosomal biomarkers for ILD. **A** Exploratory proteomic workflow for the discovery set. **B** NTA and NanoFCM analyses of serum exosomes isolated by UC and Exo-CMDS. **C** Volcano plot showing significantly upregulated (red) and downregulated (blue) proteins (FDR < 0.05). **D** Venn diagrams showing differential overlaps in ILD vs HC and ILD vs DC in the discovery set for up-regulated (top) and down-regulated (bottom) DEPs, respectively. **E** KEGG pathway enrichment analysis of up-regulated DEPs in ILD vs HC group. **F** Heatmap of 44 candidate DEPs in the HC, DC, and ILD groups (left). Log2FC and its adjusted *p*-value (p_adj_) shown for DEPs in ILD vs HC and ILD vs DC group comparisons (right). Differences between the two groups were compared using the two-tailed Mann–Whitney U-test (unadjusted): *p < 0.05, **p < 0.01, ***p < 0.001, and ****p < 0.0001. DEPs were identified according to the |log2FC|> 1.0 and *p*-value < 0.05 thresholds. CMDS, centrifugal microfluidic disc system; DC, disease control; DEPs, differentially enriched proteins; FC, fold change; FDR, false discovery rate; HC, healthy control; exo, exosome; ILD, interstitial lung disease; KEGG, Kyoto encyclopedia of genes and genomes; MS, mass spectrometry; NTA, nanoparticle tracking analysis; NanoFCM, nanoparticle flow cytometry; UC, ultracentrifugation; SE, standard error

Exosomes from 168 serum samples (discovery set: ILD, *n* = 90; HC, *n* = 31; DC, *n* = 47) were extracted using the Exo-CMDS system [23] and subjected to MS-based proteomic analysis (Fig. 1A and Fig. S1C). The Exo-CMDS yielded higher numbers of exosomes, which were more uniform in size (30–200 nm), than ultracentrifugation, the gold standard for exosomal isolation in the laboratory (Fig. 1B and Fig. S1A–B). According to the criteria (log2FC > 1.0, *p*-value < 0.05), 347 proteins (138 upregulated, 209 downregulated) among the 2,064 qualifying proteins were differentially expressed in the ILD versus HC groups, and 195 proteins (84 upregulated, 111 downregulated) in the ILD versus DC groups (Fig. 1C and Table S2–3). Of these DEPs, 51 exist in both comparison groups (Fig. 1D). Kyoto Encyclopedia and Genes and Genomes (KEGG) enrichment analysis showed that ILD-related proteins were mainly associated with the proteasome complex, extracellular-matrix-receptor interactions, focal adhesion, and platelet activation (Fig. 1E and Fig. S1D). Following the exclusion of seven proteins with low MS intensity, 44 proteins were conducted for further selection process. Then, the top 10 DEPs with the most significance were selected for further consideration (Fig. 1F, marked in red). These proteins were then subjected to a logistic regression (LR) analysis, where potential confounding factors, such as age, gender, and smoking status were included (Table S4). Finally, nine candidates with a power > 0.95 were selected as final biomarkers (Table S5).

Furthermore, we evaluated the expression profiles of candidate genes within lung tissue by using publicly available single cell sequencing datasets [24] (Fig. S2A). The results showed that ZBTB8OS, RAB3GAP2, and CFHR4 gene expressions were negligible in lung tissue and were therefore excluded from further analysis. Meanwhile, ARPC1B, ACTA2, and MYLK were found to be involved in the formation of the cytoplasmic actin network but they are not lung tissue-specific markers. Most vascular diseases can cause false positive expression. Conversely, SP-B (Surfactant Protein B, or SFTPB), CAPN2 (Calpain-2 catalytic subunit), and MUC1 (Mucin-1) emerged as compelling candidates due to their distinctive roles and expression patterns within the lung. Specifically, SP-B was found to be uniquely expressed in the lung and enriched in epithelial cells, playing a critical role in lung maintenance and repair [25]. Similarly, MUC1 is prominently expressed in lung epithelial cells and its glycoprotein subtype KL-6 has been established as a diagnostic marker for pulmonary fibrosis [26, 27]. Lastly, while CAPN2 showed a dispersed expression pattern, it was notably activated in epithelial cells, and calpain signaling pathways involving CAPN2 have been implicated in the development of pulmonary fibrosis [28]. Given this, we selected SP-B, CAPN2, and MUC1 for further analysis. Further details relating to the biomarker selection process are outlined in Fig S1E.

### Exosomal biomarkers have a stronger ability than that of plasma free proteins in distinguishing between the ILD and HC groups

**Fig. 2:**
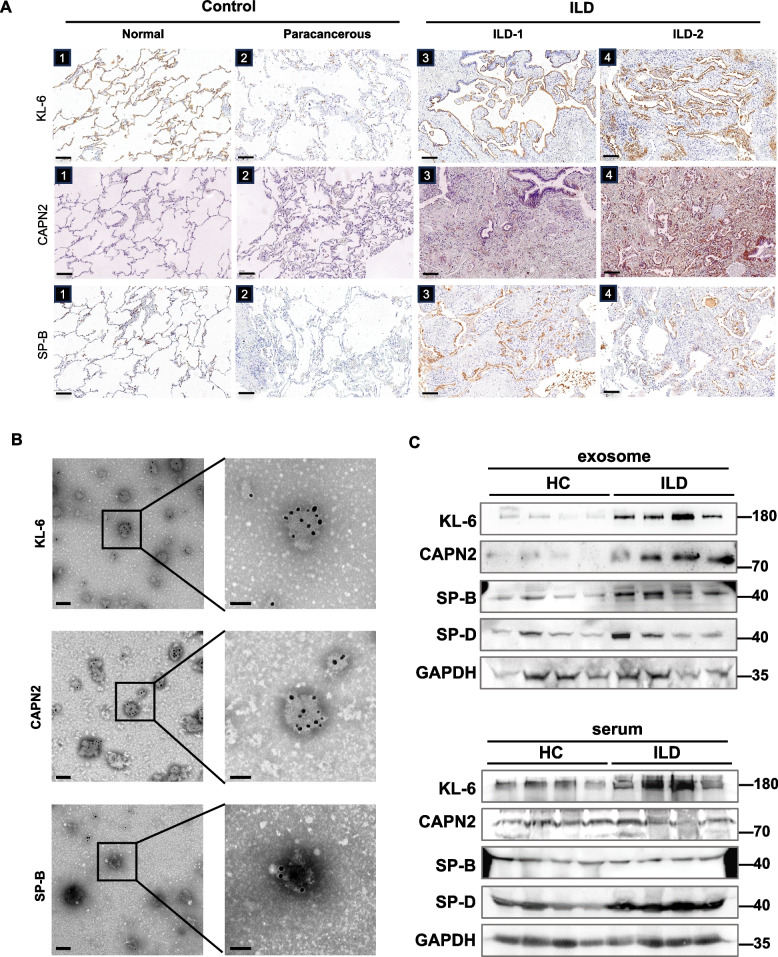
Elevated levels of KL-6, CAPN2, and SP-B in serum exosomes and lung tissue of patients with ILD. **A** Representative immunohistochemistry images of KL-6, CAPN2, and SP-B staining in normal lung tissue from a healthy donor (A1; 20 × magnification), paracancerous tissue from a lung cancer patient (A2), and lung tissue from patients with ILD (A3–A4). Samples were observed at 20 × magnification; scale bars = 100 μm. **B** Representative gold nanoparticle TEM images of KL-6, CAPN2, and SP-B in exosomal fractions isolated from serum. Scale bars = 200 nm (left) and 100 nm (right). **C** Representative immunoblots of selected biomarkers and SP-D in serum exosomes and total serum subfractions of patients with ILD and healthy individuals. ELISA, enzyme-linked immunosorbent assay; TEM, transmission electron microscopy

To ascertain the potential clinical value of these three markers, we tested their levels in a small number of randomly selected samples (ILD *n* = 9, HC *n* = 8) from the discovery set by ELISA (Fig. S3A). The results revealed significant differences in the CAPN2 and KL-6 levels between serum and blood-derived exosomal fractions. In the case of SP-B, the exosomal samples had a stronger ability than the serum counterparts to distinguish between the ILD and HC groups (Fig. S3A). Immunohistochemical analysis revealed that the lung tissues of patients with ILD expressed higher levels of KL-6/CAPN2/SP-B than those of healthy donors and the paracancerous tissues of patients with lung cancer. KL-6 and SP-B expression was prominent in alveolar cells, while CAPN2 expressions were more diffuse within the lung tissue (Fig. 2A). Cytoplasmic immunofluorescence analysis revealed significant colocalization of KL-6 and SP-B with SP-C (a specific marker of alveolar type II cells), whereas CAPN2 only partially colocalized with this marker (Fig. S3B). Moreover, immunogold transmission electron microscopy (TEM) showed that the three exosomal biomarkers localized to the membrane of exosomes derived from the serum of patients with ILD (Fig. 2B). Additionally, variations in the levels of the three biomarkers in serum and exosomal fractions of the different sample groups were detected by western blotting. The expression levels of KL-6/CAPN2/SP-B in exosomes of the ILD group were significantly higher than those of the HC group. Meanwhile, KL-6 was the only biomarker that exhibited significantly different expression levels in the serum samples of these two groups. Conversely, the expression of hydrophilic SP-D in the serum but not in exosomes differed significantly between the ILD and HC groups (Fig. 2C). Interestingly, the full-length proSP-B (42 kDa) but not its mature version (7 kDa), was readily detected in both the serum and the exosomal extract. Since proSP-B is primarily produced and processed by alveolar type II (AT2) cells, this observation indicates that there could be an abnormality in the intracellular processing mechanisms within these cells.

### Comparing the performance of exosomal KL-6/CAPN2/SP-B in the discovery and validation sets

**Fig. 3:**
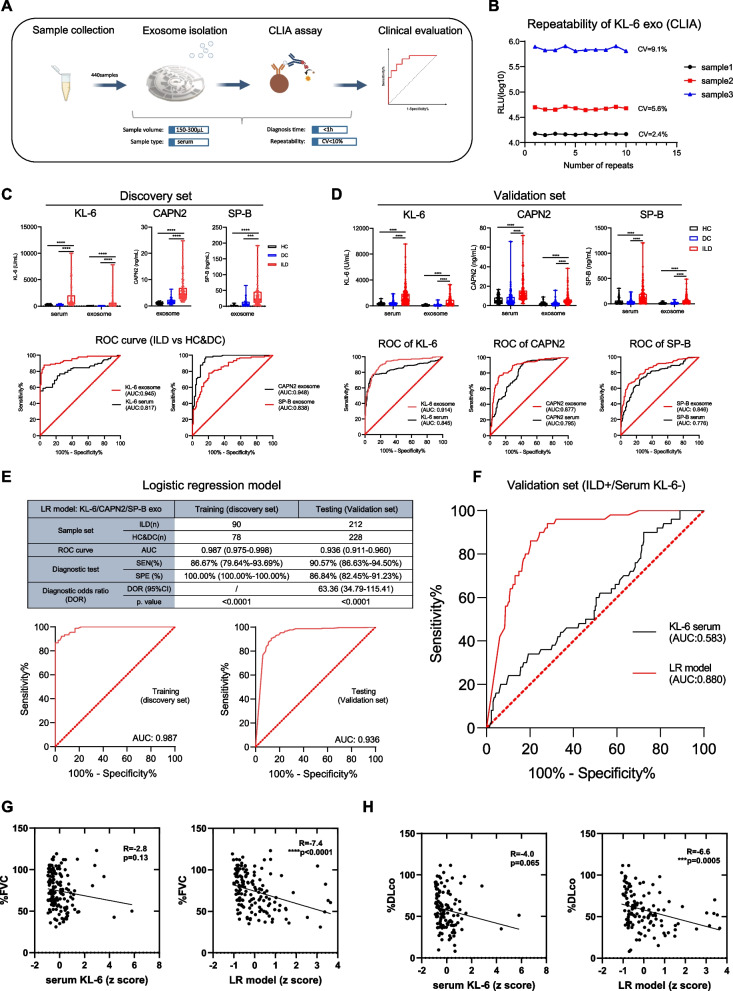
Evaluation of selected exosomal biomarkers using the Exo-CMDS and CLIA detection system. **A** Schematic representation of the Exo-CMDS- and CLIA-based exosomal protein detection system. **B** The CLIA KL-6 detection kit was used to determine exosomal KL-6 protein levels; each sample was assayed ten times. The coefficient of variation (CV) was calculated using the formula: CV = (SD/mean) × 100%. **C **and **D** The protein levels (measured using in-house KL-6/CAPN2/SP-B CLIA reagents) and corresponding ROC curves of exosomal KL-6/CAPN2/SP-B in the discovery set, which contained 31 HC, 47 DC, and 90 ILD samples (**C**), and in the validation set, which contained 93 HC, 135 DC, and 212 ILD samples (**D**). Participant serum KL-6 levels in discovery set and validation set were verified using a commercial KL-6 kit before enrollment. Levels of serum CAPN2 and SP-B in the discovery set were not assessed because most of the samples had been exhausted in previous experiments. **E** LR model performance in the training (discovery) and testing (validation) sets, regardless of demographic factors. **F** Testing score and ROC curves of the LR model of serum-KL-6-negative ILD samples in the validation set. The sigmoid function (σ[x] = 1/[1 + exp(-x)]) was used to calculate the LR model score. Differences between two groups were compared using the two-tailed Mann–Whitney U-test (unadjusted). **G **and **H** Correlations of serum KL-6 and LR model with percentage predicted forced vital capacity (%FVC) and the diffusing capacity for carbon monoxide (%DLco) using samples from validation set. *p < 0.05, **p < 0.01, ***p < 0.001, and ****p < 0.0001. ROC, receiver operating characteristic; AUC, area under the ROC curve; CLIA, chemiluminescence immunoassay; LR, logistic regression; SEN, sensitivity; SPE, specificity

We next established a chemiluminescent detection system (a combination of Exo-CMDS exosome extraction and CLIA methods) to examine the actual performance of the selected biomarkers (Fig. 3A). Reproducibility (coefficient of variation < 10%) of the system was evaluated to ensure that it was suitably robust for clinical use (Fig. 3B). According to the receiver operating characteristic (ROC) curve analysis, all three biomarkers exhibited excellent performance (area under the ROC curve [AUC] for KL-6: 0.945, 95% confidence interval [CI] 0.910–0.979; CAPN2: 0.948, 95% CI 0.915–0.981; SP-B: 0.838, 95% CI 0.778–0.898) in distinguishing the ILD group from the two control groups. In addition, exosomal KL-6/CAPN2/SP-B displayed significantly better performance at diagnosing ILD than serum KL-6 (AUC = 0.817, 95% CI 0.753–0.882; Fig. 3C). To enhance the diagnostic capacity of the markers, we next generated an LR model based on three exosomal biomarkers using the discovery set (training set) and evaluated its performance in validation set (testing set). An LR model combining exosomal KL-6/CAPN2/SP-B identified ILD in the training set independently of HC and DC with a high degree of accuracy (AUC: 0.987, 95% CI 0.975–0.998). The final LR formula was: ILD score = (0.0362 × KL-6 exo) + (1.067 × CAPN2 exo) + (0.035 × SP-B exo) – 6.54, where exo = exosomal levels.

Next, the clinical performance of individual biomarkers and the LR model were compared in a multicenter validation set with 440 samples (ILD, *n* = 212; HC, *n* = 93; DC, *n* = 135). ROC curve analysis revealed that the AUC of serum KL-6 was 0.845 (95% CI 0.805–0.885), with a sensitivity of 76.89%, specificity of 89.04%. The cut-off value for exosomal KL-6 was set at 111.4 U/mL, with a sensitivity of 87.74%, specificity of 81.58%, and AUC of 0.914 (95% CI 0.887–0.941). The cut-off values for exosomal CAPN2 and SP-B were 3.13 ng/mL and 20.15 ng/mL, with AUC of 0.877 (95% CI 0.844–0.910) and 0.846 (95% CI 0.809–0.882), both performed better than their serum counterparts (Fig. 3D and Table S6). Furthermore, the resulting ROC curve of the LR model generated an AUC of 0.935 (95% CI 0.911–0.960) in the validation set, with a sensitivity of 90.57% and a specificity of 86.84% when cutoff of ILD score was set at 0.825. The clinical performance of the LR model was significantly better than that of serum KL-6 and that of the three individual exosomal biomarkers (Fig. 3E and Table S6).

To assess whether incorporating clinical and demographic factors, such as age, sex, and serum KL-6, could impact the diagnostic power of our established LR model, these variables were incorporated into a multivariable LR analysis. The results showed that inclusion of these additional clinical parameters did not significantly influence the model's performance, suggesting that the model’s diagnostic capacity arises primarily from the three exosomal biomarkers (Fig. S4A). We next evaluated the diagnostic performance of the exosomal biomarkers and the LR model on KL-6-negative serum samples from patients with ILD, as this population is often misdiagnosed. Exosomal KL-6, CAPN2, and SP-B all clearly distinguished the KL-6-negative serum samples of the ILD group from those of the HC and DC groups (Fig. S4B). Moreover, the LR model yielded an AUC of 0.880 (95%CI 0.834–0.925) with a sensitivity of 70.00% and a specificity of 86.84%, confirming that it had robust diagnostic accuracy (Fig. 3F). Consistently, we evaluated the correlation between biomarker levels and lung function. Exosomal KL-6 showed significantly stronger correlations with both percentage of forced vital capacity (%FVC, *r* = −5.5, **p < 0.01) and percentage of diffusion capacity of the lung for carbon monoxide (%DLco, *r* = −4.7, *p < 0.05) compared to serum KL-6 (%FVC: *r* = −2.8, *p* > 0.05; %DLco: *r* = −4.0, *p* > 0.05). Although exosomal CAPN2 and SP-B showed no significant correlations (Fig. S4C-D), the LR model exhibited even stronger correlations with %FVC (*r* = −7.4, ****p < 0.0001) and %DLco (*r* = −6.6, **p < 0.001) (Fig. 3G-H). These findings demonstrate the potential role of the LR model in diagnosing early ILD and assessing lung severity.

### The potential of exosomal KL-6/CAPN2/SP-B to distinguish between CTD-ILD in CTD

**Fig. 4:**
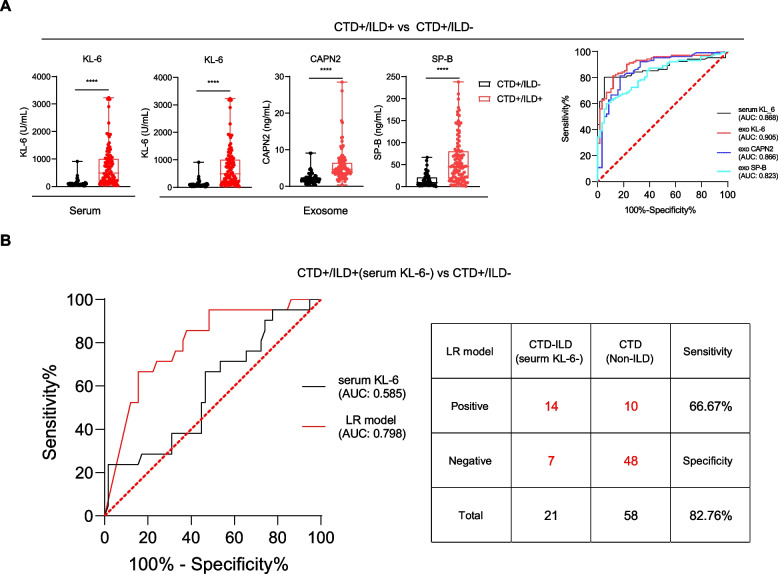
Efficiency of candidate biomarkers and LR model in distinguishing CTD from CTD-ILD. **A** Quantification and ROC curves of serum KL-6, exosomal KL-6, CAPN2, and SP-B in CTD +/ILD − (*n* = 58) and CTD +/ILD + samples (*n* = 102). Data are expressed as median (IQR). Differences between the two groups were compared by a two-tailed Mann–Whitney U-test (unadjusted): *p < 0.05, **p < 0.01, ***p < 0.001 and ****p < 0.0001. **B** ROC curve analysis of candidate biomarkers and LR model for CTD +/ILD − and serum KL-6-negative CTD + ILD +. ROC, receiver operating characteristic

CTD-ILD is a common pulmonary complication in patients with CTD, which can progress to pulmonary fibrosis, severely impairing lung function [16]. Thus, we next evaluated the performance of exosomal biomarkers in the clinical diagnosis of CTD-ILD using samples from validation set. The results revealed significant differences in the levels of serum KL-6 and exosomal KL-6/CAPN2/SP-B between the CTD^+^/ILD^−^ and CTD^+^/ILD^+^ individuals. The AUC values for all four biomarkers were > 0.8, confirming their efficacy in identifying ILD in the context of CTD (Fig. 4A). Furthermore, exosomal KL-6, CAPN2 and SP-B levels could effectively differentiate between CTD^+^/ILD^−^ and serum-KL-6-negative CTD^+^/ILD^+^, whereas serum KL-6 levels could not (Fig. S5A-B). Subsequently, the LR model analysis results (AUC = 0.798, 95%CI 0.690–0.910) showed that this biomarker combination differentiated between CTD-ILD and CTD in serum-KL-6-negative patients more effectively than serum KL-6 and the three individual exosomal biomarkers (Fig. 4B). The diagnostic efficacy of the exosomal biomarkers was then verified using HRCT by experienced clinicians (Fig. S6). Among the six CTD cases included in the analysis, two (patients B and E) were diagnosed as having CTD-ILD using the exosomal biomarkers, although their serum KL-6 results were negative. These results confirm the validity of the LR model in the diagnosis of CTD-ILD in serum-KL-6-negative patients. Based on our research, we proposed a preliminary algorithm for screening and follow-up of patients with suspected ILD or those at high risk of the disease (Fig. S7).

## Discussion

Finding a method for the precise, non-invasive, and early supplement diagnosis of ILD remains a major challenge. In this study, we used our previously developed efficient and affordable Exo-CMDS system [23] and high-throughput proteomic sequencing to identify and validate three circulating exosomal protein (KL-6, CAPN2, SP-B) for ILD in two sample sets of > 600 subjects combined. We demonstrated the feasibility of analyzing exosomal proteins in raw biological fluid samples, enabling the translation of the ILD diagnostic method from the laboratory to the clinic. To our knowledge, this is the first time a fully quantitative system for estimating protein concentrations in circulating exosomes has been established.

Normally speaking, the practical feasibility of exosomal isolation seems more complex and costly to replace serum biomarker sampling. The long processing times, high cost, and user errors have limited the interrogation of exosomes as sources of biomarkers in the clinic. Our team developed exosome isolation chip and the batch production is stable and the average cost is less than one dollar per sample. It is the key point that this technology can be implemented in clinical testing. In 2022, Bowman et al. used a 368-protein-targeting proteomic platform to identify new plasma proteomic biomarkers of progressive fibrosing ILD [29]. Although this ultra-high-sensitivity detection method can be used to find variations in trace proteins, the high cost and overly sensitive detection have restricted clinical application. To aid the discovery of biomarkers for ILD, we have established the biggest (a total of 168 samples and 2,064 proteins) human exosome protein MS database of samples from patients with ILD and control subjects to date, enabling a more accurate investigation of the disease process. Fortunately, the minimum amount of protein typically isolated from exosomes (nanogram level) is sufficient for the detection of most protein biomarkers.

KL-6, a glycoprotein subtype of MUC1, which is secreted by regenerating type II pneumocytes, has been widely used in the clinical diagnosis of ILD over the past 10 years. Previously, the N-terminal fragment of KL-6/MUC1 (MUC1-N) was thought to be released into circulation from the membranes of stressed cells [26, 27, 30]. The results of the present study indicated that the intact form of KL-6/MUC1 was also released into circulation via exosomes. Moreover, we found that exosomal KL-6/MUC1 was a more sensitive indicator of ILD than serum KL-6, which is likely because the expression of the different KL-6/MUC1 subtypes masks the expression of the subtype specifically associated with ILD. The second ILD-associated biomarker SP-B, is a hydrophobic member of the collectin protein family, which plays a vital role in regulating the surface tension of the alveoli, as well as surfactant secretion and structure [31]. Intriguingly, although the hydrophilic collectin family members SP-A and SP-D have been readily studied in ILD subjects, the diagnostic value of blood SP-B levels has not been previously recognized, possibly because its hydrophobic properties make it difficult to detect. SP-B ensures the rapid transfer of lung surfactant to the gas exchange interface, which is required for normal lung function. This rapid transfer is likely achieved via a hemi-fusion stalk connection between the interface and surfactant vesicles/reservoirs [32, 33]. The SP-B protein has been detected in exosomes extracted from human lung cancer cells and malignant pleural effusions [34]. It seems that hydrophobic polypeptides participate in the fusion of lipid vesicles (e.g., exosomes) than hydrophilic ones, meaning that they are easier to detect than free protein. Here, we used MS and western blotting to confirm that exosomes mainly contained full-length proSP-B rather than its mature form, offering new perspectives on the function of pulmonary surfactant proteins during lung injury. The final biomarker CAPN2 is the catalytic subunit of calpain-2. Serum calpain activity was reported as an independent risk factor for systemic-sclerosis-associated-ILD [35]. Moreover, the calpain inhibitor calpeptin prevented bleomycin-induced pulmonary fibrosis in mice, suggesting the potential involvement of calpain in ILD development [28]. High calpain activity is also associated with the release of inflammatory mediators, increased apoptosis, and abnormal MMP activation, all of which may induce pulmonary fibrosis [36, 37].

Despite the identification of multiple diagnostic and prognostic indicators of ILD, no single indicator has performed perfectly in terms of sensitivity, specificity, and accuracy. Moreover, although the serum levels of other biomarkers (e.g., MMP-7 and SP-D) were significantly raised in patients with IPF [6], until now, no new biomarkers for ILD outperformed serum KL-6 in the clinic. In the study, exosomal KL-6 and CAPN2 levels both displayed better diagnostic performance than serum KL-6 levels. It must be noted, however, that the overall clinical sensitivity of serum KL-6 was lower in our study than in previous reports. The reason for this discrepancy may be that we specifically included samples from serum-KL-6-negative ILD patients (41 in the discovery set and 50 in the validation set) in our study. The inclusion of these samples highlighted the value of exosome biomarkers in the detection of serum-KL-6-negative serum ILD samples. Moreover, although our investigation uncovered several other exosomal biomarkers (e.g., von Willebrand factor [vWF], vitamin K-dependent protein C, and numerous proteasome subunits) that effectively discriminated between the ILD and HC groups, their inability to accurately distinguish ILD from DC led to their exclusion from further analysis. Indeed, raised vWF levels are not specific to ILD and are consistently observed across a range of diseases such as cholangiocarcinoma [38], lung cancer [39], and oral squamous cell carcinoma [40]. The non-specific nature of exosomal biomarkers such as vWF justifies our strategy of concurrently assessing biomarker levels in ILD versus both HC and DC groups.

CTD-ILD is a common and serious complication of CTD, which is characterized by pulmonary inflammation and interstitial fibrosis. HRCT has previously revealed that 65% of patients with scleroderma and 60% of patients with rheumatoid arthritis had ILD [41]. The peripheral blood biomarkers for CTD-ILD identified to date, however, are not sufficiently diagnostically accurate and are therefore not routinely used in clinical practice. The combination of exosomal SP-B&CAPN2 was able to distinguish CTD-ILD from the control groups when serum KL-6 levels were undetectable; no existing CTD-ILD biomarkers can rival this level of accuracy. Thus, although further mechanistic and clinical studies are needed, exosomal biomarkers may serve as a valuable tool for obtaining a clinical diagnosis of CTD-ILD.

Despite reporting important results, our study had several limitations. First, the observed concentrations of CAPN2 were relatively low, necessitating the development of new antibody pairs. Second, the pool of clinical samples used for testing was insufficiently large. Future research should aim to validate the identified biomarkers in a more diverse patient population, ideally drawn from multiple centers across different countries. Third, we did not document the specific pharmacotherapeutic regimens of the enrolled subjects at the time of sampling, which made it difficult to determine whether our results were confounded by patient medication. Besides, our results warrant further mechanistic exploration to better understand the underlying processes. Addressing these shortcomings in subsequent prospective studies will be crucial for refining the diagnostic model and ensuring its clinical applicability.

Our study highlighted the value of the Exo-CMDS-based automated chemiluminescent detection system as a reproducible and efficient means of analyzing exosomal biomarkers. Using this system, we successfully identified exosomal KL-6, CAPN2, and SP-B levels as promising diagnostic biomarkers for ILD. The individual diagnostic efficacies of these biomarkers were further increased by their inclusion in a multivariable LR model. The clinical implications of our findings will be explored in future studies.

## Supplementary Information

Supplementary Material 1.

Supplementary Material 2.

Supplementary Material 3.

## Data Availability

The data that support the findings of this study are not openly available due to reasons of sensitivity and are available from the corresponding author upon reasonable request.

## References

[1] Ageely G, Souza C, De Boer K, Zahra S, Gomes M, Voduc N. The Impact of Multidisciplinary Discussion (MDD) in the Diagnosis and Management of Fibrotic Interstitial Lung Diseases. Can Respir J. 2020;2020:9026171. 32879642 10.1155/2020/9026171 PMC7448233

[2] Hoyer N, Prior TS, Bendstrup E, Wilcke T, Shaker SB. Risk factors for diagnostic delay in idiopathic pulmonary fibrosis. Respir Res. 2019;20:103. 31126287 10.1186/s12931-019-1076-0 PMC6534848

[3] Cosgrove GP, Bianchi P, Danese S, Lederer DJ. Barriers to timely diagnosis of interstitial lung disease in the real world: the INTENSITY survey. BMC Pulm Med. 2018;18:9. 29343236 10.1186/s12890-017-0560-x PMC5773175

[4] Biglia C, Ghaye B, Reychler G, Koenig S, Yildiz H, Lacroix V, Tamirou F, Hoton D, Pieters T, Froidure A. Multidisciplinary management of interstitial lung diseases: A real-life study. Sarcoidosis Vasc Diffuse Lung Dis. 2019;36:108–15. 32476943 10.36141/svdld.v36i2.8107 PMC7247099

[5] Oldham JM, Adegunsoye A, Khera S, Lafond E, Noth I, Strek ME, Kadoch M, Chung JH. Underreporting of Interstitial Lung Abnormalities on Lung Cancer Screening Computed Tomography. Ann Am Thorac Soc. 2018;15:764–6. 29490147 10.1513/AnnalsATS.201801-053RL PMC6137678

[6] Hamai K, Iwamoto H, Ishikawa N, Horimasu Y, Masuda T, Miyamoto S, Nakashima T, Ohshimo S, Fujitaka K, Hamada H, et al. Comparative Study of Circulating MMP-7, CCL18, KL-6, SP-A, and SP-D as Disease Markers of Idiopathic Pulmonary Fibrosis. Dis Markers. 2016;2016:4759040. 27293304 10.1155/2016/4759040 PMC4886062

[7] Lee JS, Lee EY, Ha YJ, Kang EH, Lee YJ, Song YW. Serum KL-6 levels reflect the severity of interstitial lung disease associated with connective tissue disease. Arthritis Res Ther. 2019;21:58. 30764869 10.1186/s13075-019-1835-9 PMC6376648

[8] Yanaba K, Hasegawa M, Takehara K, Sato S. Comparative study of serum surfactant protein-D and KL-6 concentrations in patients with systemic sclerosis as markers for monitoring the activity of pulmonary fibrosis. J Rheumatol. 2004;31:1112–20. 15170923

[9] Tzouvelekis A, Kouliatsis G, Anevlavis S, Bouros D. Serum biomarkers in interstitial lung diseases. Respir Res. 2005;6:78. 16042760 10.1186/1465-9921-6-78 PMC1215520

[10] Chen G, Huang AC, Zhang W, Zhang G, Wu M, Xu W, Yu Z, Yang J, Wang B, Sun H, et al. Exosomal PD-L1 contributes to immunosuppression and is associated with anti-PD-1 response. Nature. 2018;560:382–6. 30089911 10.1038/s41586-018-0392-8 PMC6095740

[11] Yang D, Zhang W, Zhang H, Zhang F, Chen L, Ma L, Larcher LM, Chen S, Liu N, Zhao Q, et al. Progress, opportunity, and perspective on exosome isolation - efforts for efficient exosome-based theranostics. Theranostics. 2020;10:3684–707. 32206116 10.7150/thno.41580 PMC7069071

[12] Qazi KR, Torregrosa Paredes P, Dahlberg B, Grunewald J, Eklund A, Gabrielsson S. Proinflammatory exosomes in bronchoalveolar lavage fluid of patients with sarcoidosis. Thorax. 2010;65:1016–24. 20880880 10.1136/thx.2009.132027

[13] Martinez-Bravo MJ, Wahlund CJ, Qazi KR, Moulder R, Lukic A, Radmark O, Lahesmaa R, Grunewald J, Eklund A, Gabrielsson S. Pulmonary sarcoidosis is associated with exosomal vitamin D-binding protein and inflammatory molecules. J Allergy Clin Immunol. 2017;139:1186–94. 27566455 10.1016/j.jaci.2016.05.051

[14] Wahlund CJE, Gucluler Akpinar G, Steiner L, Ibrahim A, Bandeira E, Lepzien R, Lukic A, Smed-Sorensen A, Kullberg S, Eklund A, et al. Sarcoidosis exosomes stimulate monocytes to produce pro-inflammatory cytokines and CCL2. Sci Rep. 2020;10:15328. 32948789 10.1038/s41598-020-72067-7 PMC7501276

[15] Guo B, Wang L, Xia S, Mao M, Qian W, Peng X, Zheng Z, Chen R, Han Q, Luo Q. The interstitial lung disease spectrum under a uniform diagnostic algorithm: a retrospective study of 1,945 individuals. J Thorac Dis. 2020;12:3688–96. 32802448 10.21037/jtd-19-4021 PMC7399396

[16] Kondoh Y, Makino S, Ogura T, Suda T, Tomioka H, Amano H, Anraku M, Enomoto N, Fujii T, Fujisawa T, et al. 2020 guide for the diagnosis and treatment of interstitial lung disease associated with connective tissue disease. Respir Investig. 2021;59:709–40. 10.1016/j.resinv.2021.04.011 34602377

[17] Raghu G, Remy-Jardin M, Richeldi L, Thomson CC, Inoue Y, Johkoh T, Kreuter M, Lynch DA, Maher TM, Martinez FJ, et al. Idiopathic Pulmonary Fibrosis (an Update) and Progressive Pulmonary Fibrosis in Adults: An Official ATS/ERS/JRS/ALAT Clinical Practice Guideline. Am J Respir Crit Care Med. 2022;205:e18–47. 35486072 10.1164/rccm.202202-0399ST PMC9851481

[18] Travis WD, Costabel U, Hansell DM, King TE Jr, Lynch DA, Nicholson AG, Ryerson CJ, Ryu JH, Selman M, Wells AU, et al. An official American Thoracic Society/European Respiratory Society statement: Update of the international multidisciplinary classification of the idiopathic interstitial pneumonias. Am J Respir Crit Care Med. 2013;188:733–48. 24032382 10.1164/rccm.201308-1483ST PMC5803655

[19] Fischer A, Antoniou KM, Brown KK, Cadranel J, Corte TJ, du Bois RM, Lee JS, Leslie KO, Lynch DA, Matteson EL, et al. An official European Respiratory Society/American Thoracic Society research statement: interstitial pneumonia with autoimmune features. Eur Respir J. 2015;46:976–87. 26160873 10.1183/13993003.00150-2015

[20] Shiboski CH, Shiboski SC, Seror R, Criswell LA, Labetoulle M, Lietman TM, Rasmussen A, Scofield H, Vitali C, Bowman SJ, et al. 2016 American College of Rheumatology/European League Against Rheumatism classification criteria for primary Sjogren’s syndrome: A consensus and data-driven methodology involving three international patient cohorts. Ann Rheum Dis. 2017;76:9–16. 27789466 10.1136/annrheumdis-2016-210571

[21] Barbhaiya M, Zuily S, Naden R, Hendry A, Manneville F, Amigo MC, Amoura Z, Andrade D, Andreoli L, Artim-Esen B, et al. The 2023 ACR/EULAR Antiphospholipid Syndrome Classification Criteria. Arthritis Rheumatol. 2023;75:1687–702. 37635643 10.1002/art.42624

[22] Aletaha D, Neogi T, Silman AJ, Funovits J, Felson DT, Bingham CO 3rd, Birnbaum NS, Burmester GR, Bykerk VP, Cohen MD, et al. 2010 Rheumatoid arthritis classification criteria: an American College of Rheumatology/European League Against Rheumatism collaborative initiative. Arthritis Rheum. 2010;62:2569–81. 20872595 10.1002/art.27584

[23] Zhao L, Wang H, Fu J, Wu X, Liang XY, Liu XY, Wu X, Cao LL, Xu ZY, Dong M. Microfluidic-based exosome isolation and highly sensitive aptamer exosome membrane protein detection for lung cancer diagnosis. Biosens Bioelectron. 2022;214:114487. 35780540 10.1016/j.bios.2022.114487

[24] Natri HM, Del Azodi CB, Peter L, Taylor CJ, Chugh S, Kendle R, Chung MI, Flaherty DK, Matlock BK, Calvi CL, et al. Cell type-specific and disease-associated eQTL in the human lung. bioRxiv 2023.

[25] Ding J, Takamoto DY, von Nahmen A, Lipp MM, Lee KY, Waring AJ, Zasadzinski JA. Effects of lung surfactant proteins, SP-B and SP-C, and palmitic acid on monolayer stability. Biophys J. 2001;80:2262–72. 11325728 10.1016/S0006-3495(01)76198-X PMC1301417

[26] Nath S, Mukherjee P. MUC1: a multifaceted oncoprotein with a key role in cancer progression. Trends Mol Med. 2014;20:332–42. 24667139 10.1016/j.molmed.2014.02.007 PMC5500204

[27] Ishikawa N, Hattori N, Yokoyama A, Kohno N. Utility of KL-6/MUC1 in the clinical management of interstitial lung diseases. Respir Investig. 2012;50:3–13. 10.1016/j.resinv.2012.02.001 22554854

[28] Liu Y, Liu B, Zhang GQ, Zou JF, Zou ML, Cheng ZS. Calpain inhibition attenuates bleomycin-induced pulmonary fibrosis via switching the development of epithelial-mesenchymal transition. Naunyn Schmiedebergs Arch Pharmacol. 2018;391:695–704. 29666896 10.1007/s00210-018-1499-z PMC5994212

[29] Bowman WS, Newton CA, Linderholm AL, Neely ML, Pugashetti JV, Kaul B, Vo V, Echt GA, Leon W, Shah RJ, et al. Proteomic biomarkers of progressive fibrosing interstitial lung disease: a multicentre cohort analysis. Lancet Respir Med. 2022;10:593–602. 35063079 10.1016/S2213-2600(21)00503-8 PMC9177713

[30] Curry JM, Thompson KJ, Rao SG, Besmer DM, Murphy AM, Grdzelishvili VZ, Ahrens WA, McKillop IH, Sindram D, Iannitti DA, et al. The use of a novel MUC1 antibody to identify cancer stem cells and circulating MUC1 in mice and patients with pancreatic cancer. J Surg Oncol. 2013;107:713–22. 23335066 10.1002/jso.23316 PMC3880940

[31] Whitsett JA, Weaver TE. Hydrophobic surfactant proteins in lung function and disease. N Engl J Med. 2002;347:2141–8. 12501227 10.1056/NEJMra022387

[32] Chavarha M, Khoojinian H, Schulwitz LE Jr, Biswas SC, Rananavare SB, Hall SB. Hydrophobic surfactant proteins induce a phosphatidylethanolamine to form cubic phases. Biophys J. 2010;98:1549–57. 20409474 10.1016/j.bpj.2009.12.4302 PMC2856140

[33] Rugonyi S, Biswas SC, Hall SB. The biophysical function of pulmonary surfactant. Respir Physiol Neurobiol. 2008;163:244–55. 18632313 10.1016/j.resp.2008.05.018 PMC2669693

[34] Park JO, Choi DY, Choi DS, Kim HJ, Kang JW, Jung JH, Lee JH, Kim J, Freeman MR, Lee KY, et al. Identification and characterization of proteins isolated from microvesicles derived from human lung cancer pleural effusions. Proteomics. 2013;13:2125–34. 23585444 10.1002/pmic.201200323

[35] Zheng JN, Li Y, Yan YM, Yu Y, Shao WQ, Wang Q. Increased serum calpain activity is associated with HMGB1 levels in systemic sclerosis. Arthritis Res Ther. 2020;22:110. 32393322 10.1186/s13075-020-02195-y PMC7216546

[36] Storr SJ, Carragher NO, Frame MC, Parr T, Martin SG. The calpain system and cancer. Nat Rev Cancer. 2011;11:364–74. 21508973 10.1038/nrc3050

[37] Zhang L, Zheng D, Yan Y, Yu Y, Chen R, Li Z, Greer PA, Peng T, Wang Q. Myeloid cell-specific deletion of Capns1 prevents macrophage polarization toward the M1 phenotype and reduces interstitial lung disease in the bleomycin model of systemic sclerosis. Arthritis Res Ther. 2022;24:148. 35729674 10.1186/s13075-022-02833-7 PMC9210712

[38] Lapitz A, Azkargorta M, Milkiewicz P, Olaizola P, Zhuravleva E, Grimsrud MM, Schramm C, Arbelaiz A, O’Rourke CJ, La Casta A, et al. Liquid biopsy-based protein biomarkers for risk prediction, early diagnosis, and prognostication of cholangiocarcinoma. J Hepatol. 2023;79:93–108. 36868481 10.1016/j.jhep.2023.02.027 PMC10292605

[39] Luo B, Que Z, Lu X, Qi D, Qiao Z, Yang Y, Qian F, Jiang Y, Li Y, Ke R, et al. Identification of exosome protein panels as predictive biomarkers for non-small cell lung cancer. Biol Proced Online. 2023;25:29. 37953280 10.1186/s12575-023-00223-0 PMC10641949

[40] Guo H, Jiang W, Huang S, Huang X, Li C. Serum exosome-derived biomarkers for the early detection of oral squamous cell carcinoma. Mol Cell Biochem. 2021;476:4435–47. 34468926 10.1007/s11010-021-04254-7

[41] Hyldgaard C, Hilberg O, Pedersen AB, Ulrichsen SP, Lokke A, Bendstrup E, Ellingsen T. A population-based cohort study of rheumatoid arthritis-associated interstitial lung disease: comorbidity and mortality. Ann Rheum Dis. 2017;76:1700–6. 28611082 10.1136/annrheumdis-2017-211138

