# Supplementary material for: The exosomal protein biomarkers auxiliary in diagnosis of interstitial lung disease
Source: Respir Res. 2025 Aug 1;26:255. doi: 10.1186/s12931-025-03326-2 (PMC12317503; doi:10.1186/s12931-025-03326-2)
Supplement: Supplementary file 2 — Supplementary Material 2. [file 12931_2025_3326_MOESM2_ESM.pdf]

Contents

Figure S1. Screening and selection of exosomal biomarkers for ILD.

Figure S2. Expression of the candidate biomarkers in ILD samples at a single cell level.

Figure S3. Elevated levels of KL-6, CAPN2, and SP-B in serum exosomes and lung tissue of patients with ILD.

Figure S4. Exo-CMDS based CLIA assay of selected exosomal biomarkers.

Figure S5. Efficiency of candidate biomarkers and LR model in distinguishing CTD from CTD-ILD.

Figure S6. Interstitial lung abnormalities on chest HRCT scans.

Figure S7. Proposed diagnostic algorithm for individuals with suspected ILD or at high risk of ILD.

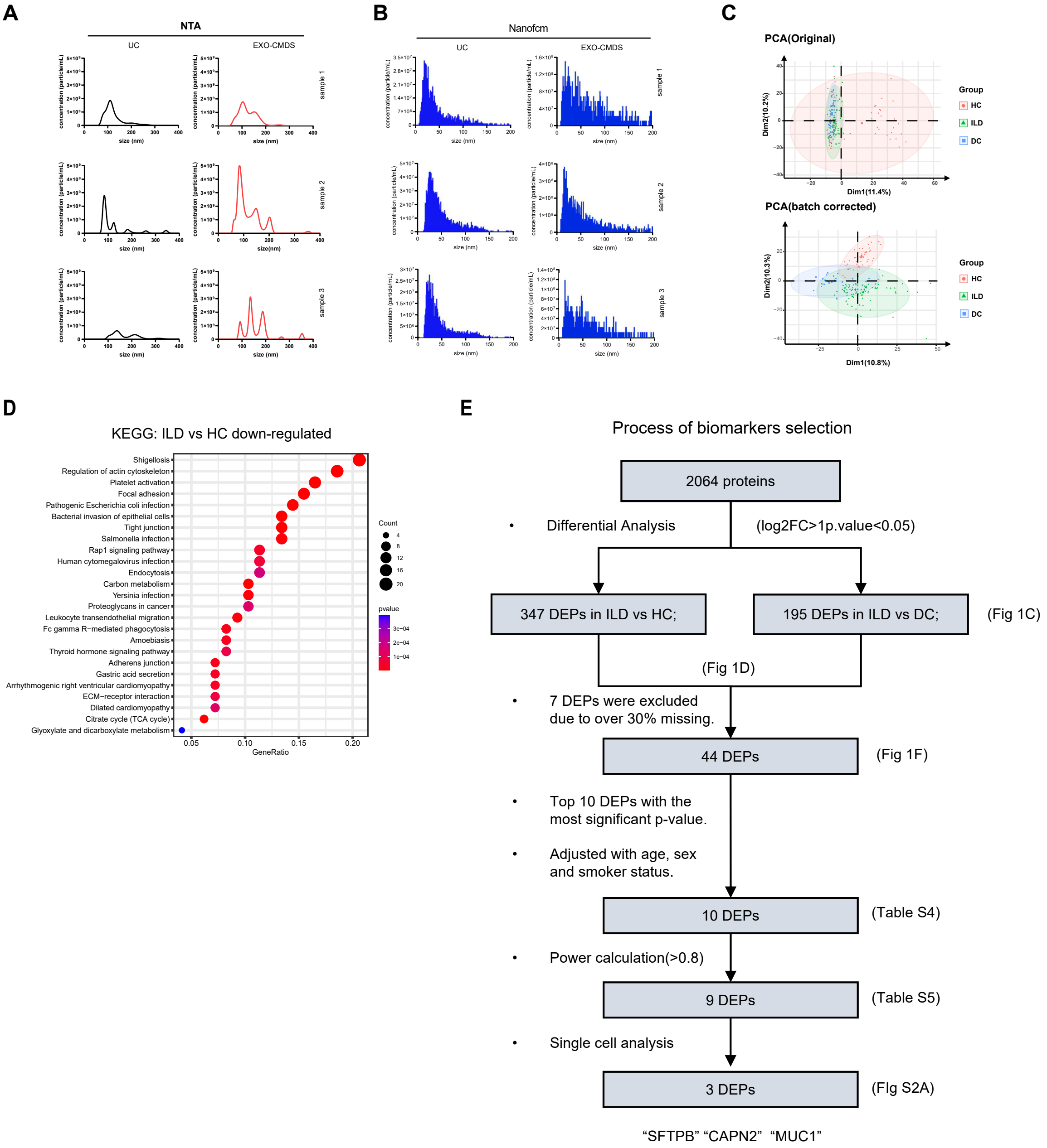

Figure S1. Screening and selection of exosomal biomarkers for ILD. (A-B) NTA **(A)** and NanoFCM **(B)** analysis of three serum exosome samples. **(C)** PCA score plot of the serum samples in the discovery set displaying separation of each group. **(D)** KEGG pathway enrichment analysis of down-regulated proteins associated in ILD vs HC group in the discovery set (FDR < 0.05). **(E)** Flow chart for the process of biomarkers selection. KEGG= Kyoto Encyclopedia of Genes and Genomes; FDR= false discovery rate; PCA=principal component analysis.

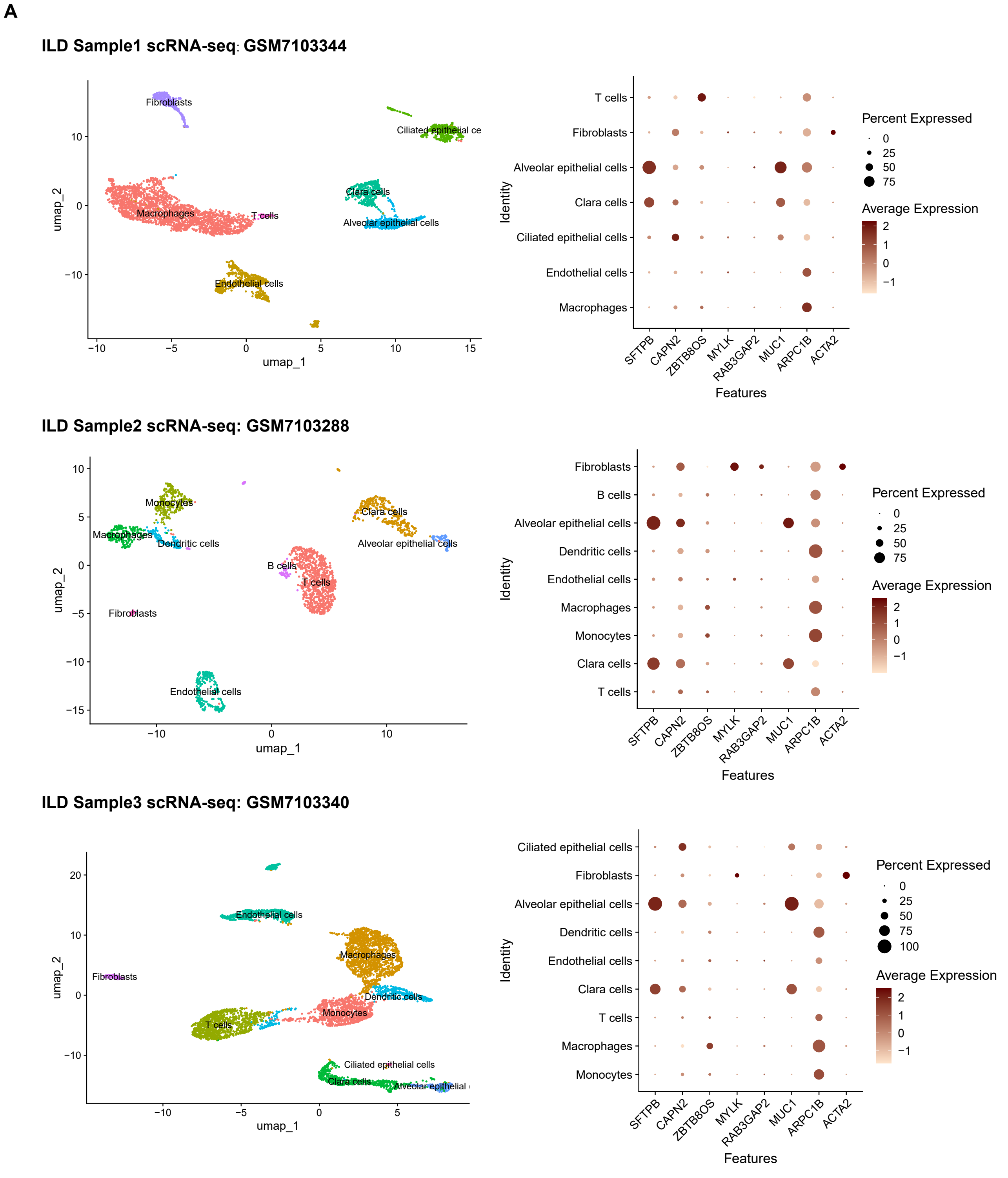

Figure S2. Expression of the candidate biomarkers in ILD samples at a single cell level. (A) UMAP plots (left) of single-cell types and dot plots (right) of candidate biomarkers across various cell types in lung tissue from Patients with ILD. Sample1: GSM7103344, Male, 65 years, diagnosed as CTD-ILD, with smoker history; Sample 2: GSM7103288, Male, 61 years, diagnosed as IPF, without smoker history; Sample 3: GSM7103340, Female, 68 years, diagnosed as IPF, with smoker history. Data about CFHR4 was missed due to extremely low expression level.

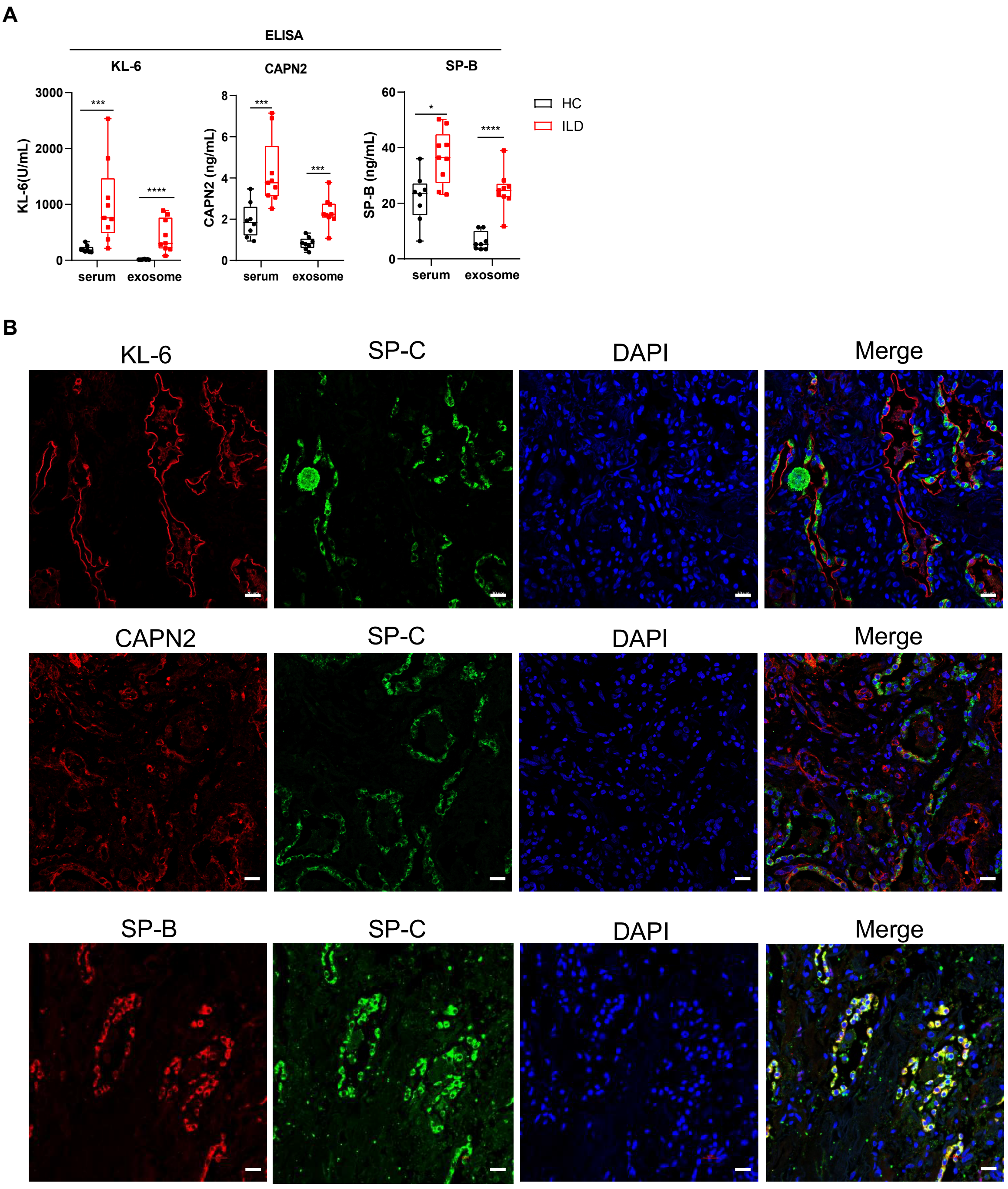

Figure S3. Elevated levels of KL-6, CAPN2, and SP-B in serum exosomes and lung tissue of patients with ILD. (A) ELISA measurement of selected biomarkers in both serum and exosomal samples from ILD and HC groups. **(B)** Paraffin sections of human ILD tissue prepared for double-label immunofluorescence and observed by laser scanning confocal microscopy (×200 magnification). ELISA= Enzyme linked immunosorbent assay.

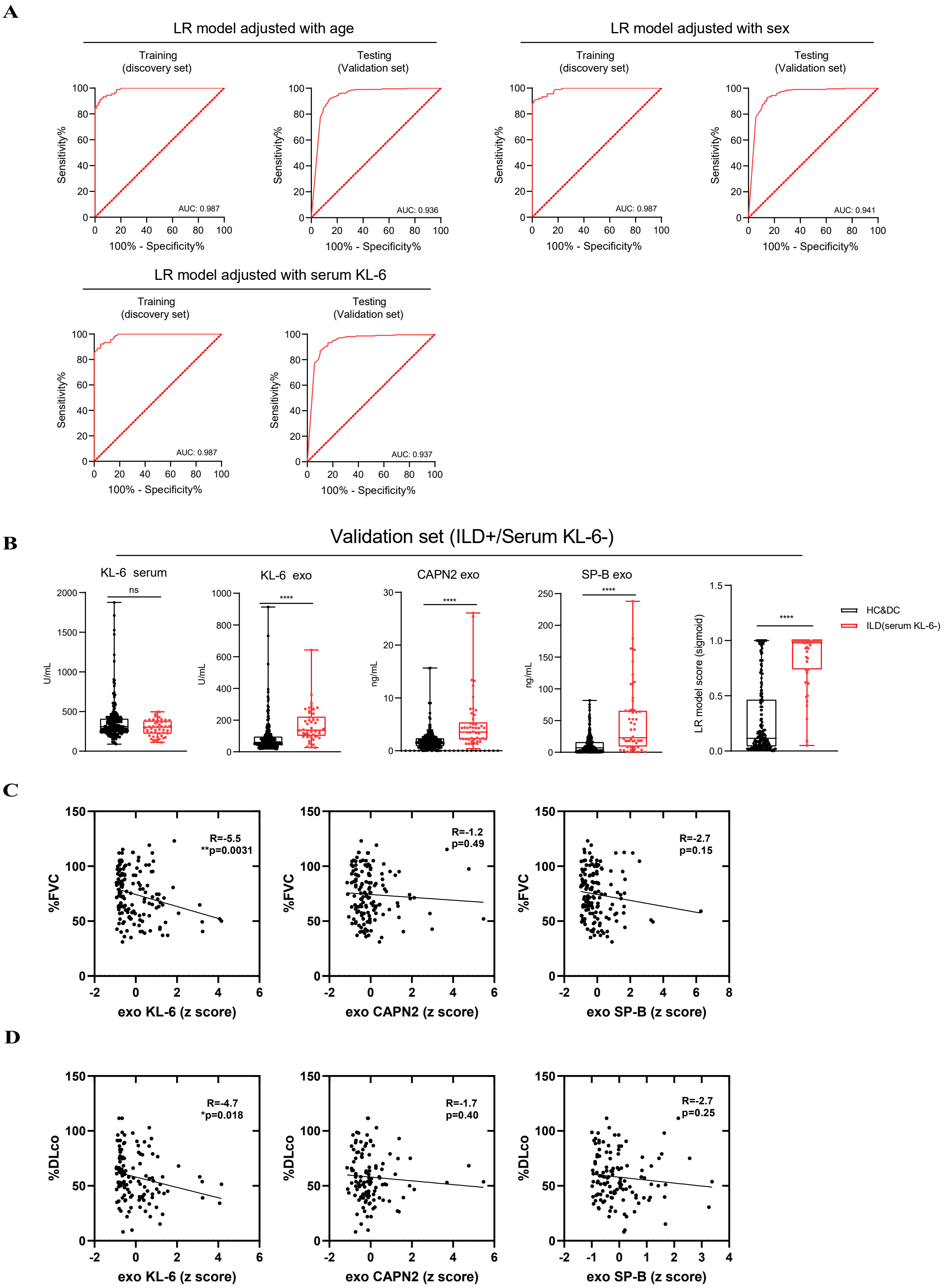

Figure S4. Exo-CMDS based CLIA assay of selected exosomal biomarkers. (A) Logistic regression models in the training (discovery) set, as well as in the testing (validation) set adjusted with demographic factors. **(B)** Levels of exosomal KL-6, CAPN2, and SP-B and score of LR model in serum-KL-6-negative ILD samples of the validation set. **(C)** Correlations of exosomal biomarkers with percentage predicted forced vital capacity (%FVC) and the diffusing capacity for carbon monoxide (%DLco) using samples from validation set. *P < 0.05, **P < 0.01, ***P < 0.001, ****P < 0.0001.

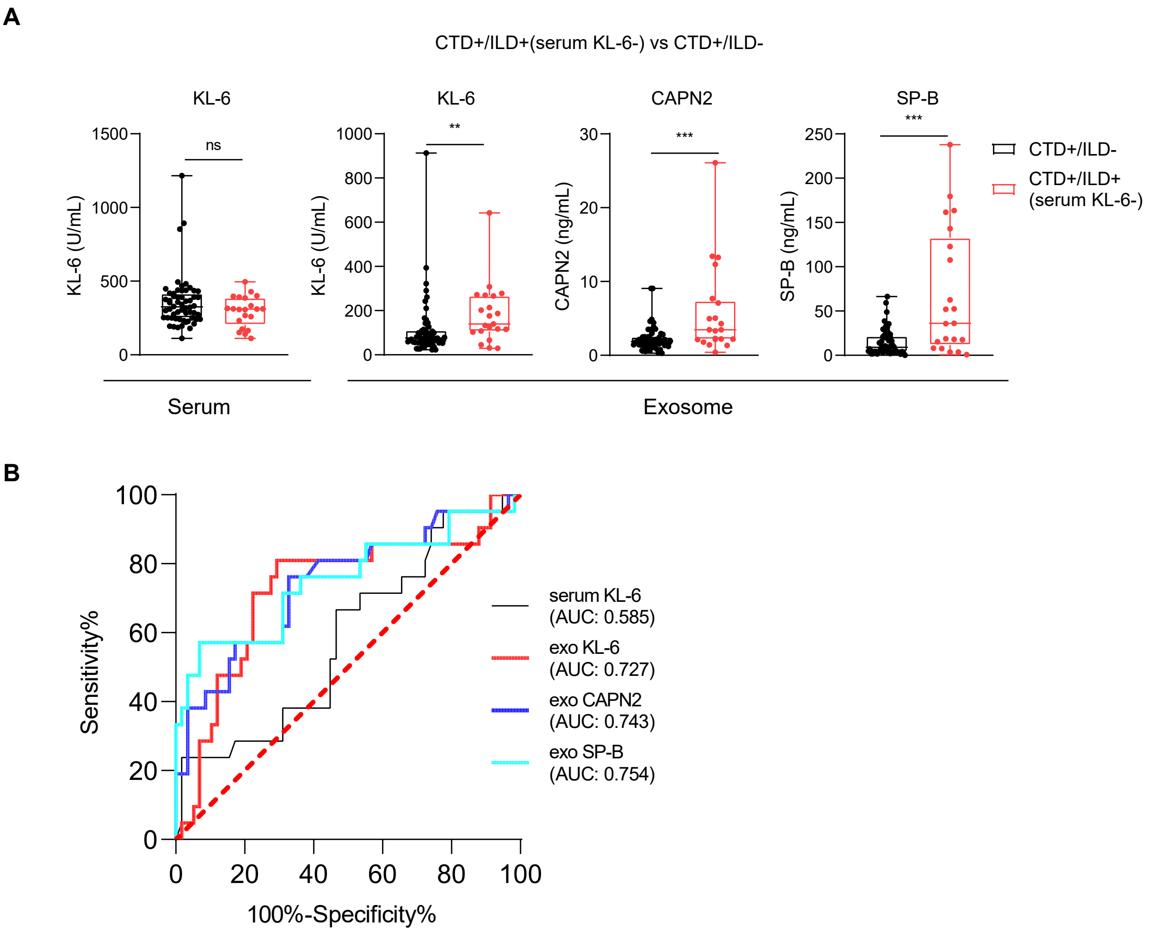

Figure S5. Efficiency of candidate biomarkers and LR model in distinguishing CTD from CTD-ILD. (A) Quantification and **(B)** ROC curves of serum KL-6, exosomal KL-6, CAPN2, and SP-B in CTD+/ILD− (n = 58) and serum KL-6-negative CTD+/ILD+ groups (n = 21)**.** Data are expressed as median (IQR).

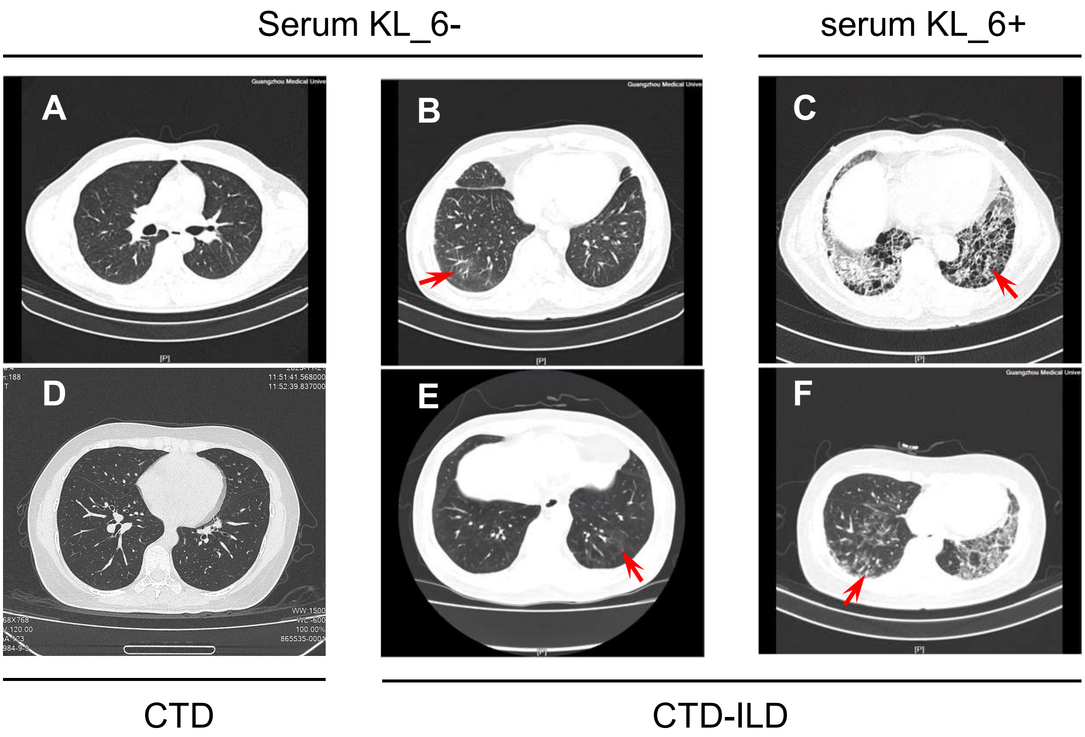

FigureS6.Interstitial lung abnormalities on chest HRCT scans
Patients diagnosed with Rheumatoid Arthritis (RA) **(A-C)**. Patients diagnosed with primary Sjögren's syndrome (pSS) **(D-F)**. Serum KL_6- without ILD, no pathological changes in the pulmonary interstitium **(A and D)**. Serum KL_6- with ILD, increased interstitial lung texture (arrow) **(B and E)**. Serum KL_6 positive with ILD, demonstrated honeycomb shadow, reticular abnormalities, and traction bronchiectasis (arrow) **(C and F)**.

Case A
A 43-year-old male smoker diagnosed with RA. The LR model predicts negative results (serum KL-6: 434 U/mL, KL-6 exo: 322.01U/mL, CAPN2: 0.77 ng/mL, exo SP-B: 6.70 ng/mL). No abnormalities were found in chest HRCT and PFTs.
Case B
A 59-year-old male smoker diagnosed with RA-ILD. The LR model predicts positive (serum KL-6: 164 U/mL, exo KL-6: 45.22U/mL, exo CAPN2: 3.31 ng/mL, exo SP-B: 15.24 ng/mL). In the PFTs, only DLco% (72%) was reduced while chest HRCT revealed GGO.
Case C
A 69-year-old male smoker diagnosed with RA-ILD. The LR model predicts positive (serum KL-6: 3123 U/mL, exo KL-6: 3177.64 U/mL, exo CAPN2: 5.07 ng/mL, exo SP-B: 114.04 ng/mL). FVC (52%) and DLco% (68%) were reduced. Chest HRCT showed a reticular pattern with honeycombing.
Case D
A 39-year-old non-smoking female was diagnosed with PSS. The LR model predicts negative (Serum KL-6: 222 U/mL, exo KL-6: 61.97 U/mL, exo CAPN2: 1.60 ng/mL, exo SP-B: 2.88 ng/mL). There was no abnormality in chest HRCT and PFTs.
Case E
A 69-year-old non-smoking female was diagnosed with PSS-ILD. The LR model predicts positive (serum KL-6: 152 U/mL, exo KL-6: 201.67 U/mL, exo CAPN2: 2.28 ng/mL, exo SP-B: 17.98 ng/mL). FVC (75%) and DLco% (50%) were reduced. HRCT showed interstitial inflammation with GGO.
Case F
A 40-year-old non-smoking female diagnosed with PSS-ILD. The LR model predicts positive (serum KL-6: 2906 U mL, exo KL-6: 2566.43 U/mL, CAPN2 exo: 3.20 ng/mL, SP-B exo: 40.56 ng/mL). FVC (65%) and DLco% (58%) were reduced. Chest HRCT was conducted and showed diffuse GGO, reticular abnormalities, and traction bronchiectasis.

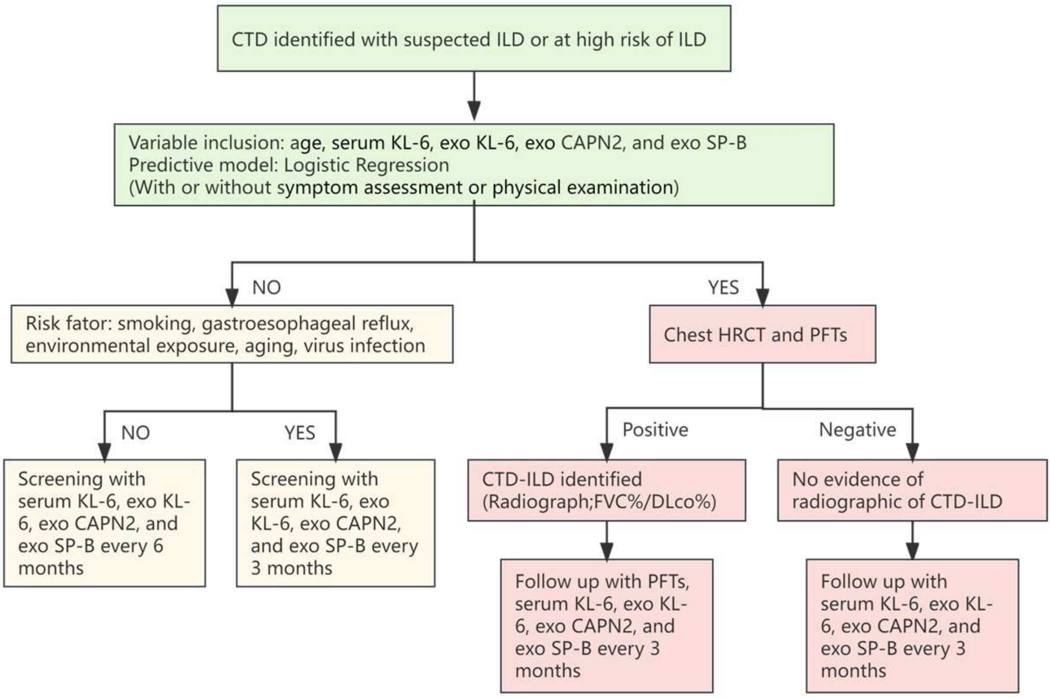

Figure S7. Proposed diagnostic algorithm for individuals with suspected ILD or at high risk of ILD

Preliminary algorithm for screening and follow-up of early CTD-ILD. CTD=connective tissue disease-associated; CTD-ILD=connective tissue disease-associated interstitial lung disease; DLco=diffusing capacity of the lungs for carbon monoxide; exo= exosome; FVC=forced vital capacity; HRCT=high-resolution CT; ILD=interstitial lung disease; PFTs=Pulmonary function tests.

1. Use the logistic regression (LR) model to analyze CTD patients for the presence of ILD early in the disease course.
2. Patients without lung involvement (with LR model predicting negative) could be monitored every 3 to 6 months (depending on the presence of risk factors for CTD-ILD) using serum KL-6, exo KL-6, exo CAPN2, and exo SP-B.
3. If there is suspicion of ILD following initial tests (with LR model predicting positive), it is recommended that the patient undergo a chest high-resolution computed tomography (HRCT) and pulmonary function tests (PFTs) to diagnose ILD and evaluate the extent of anatomical (HRCT of the chest) and physiological (PFTs) involvement.
4. Patients with confirmed radiographic ILD may undergo PFTs, clinical assessment, and analysis with LR model by screening serum KL-6, exo KL-6, exo CAPN2, and exo SP-B to every 3 to 6 months based on clinical behavior (progressive or stable).
5. If HRCT does not confirm ILD, patients are advised to be screened by serum KL-6, exo KL-6, exo CAPN2, and exo SP-B every 3 months.
