# Supplementary material for: The exosomal protein biomarkers auxiliary in diagnosis of interstitial lung disease
Source: Respir Res. 2025 Aug 1;26:255. doi: 10.1186/s12931-025-03326-2 (PMC12317503; doi:10.1186/s12931-025-03326-2)
Supplement: Supplementary file 3 — Supplementary Material 3. [file 12931_2025_3326_MOESM3_ESM.docx]

**Supplemental Methods**

**Exosome isolation and characterization**

Serum was isolated from patient peripheral blood by centrifugation for 10 min at 2,300 × g, aliquoted into polypropylene tubes, and stored at -80℃. Then 150 µl of serum was infused into the in-house Exo-CMDS chip (FDA, RE No: 3021041608) for exosomal separation as previously described^18^. The exosomes were then stored at −80 ℃ until further use. Exosomes were images by TEM (FEI, Tecnai G2 Spirit) at 80 kV. An NTA system equipped with NanoSight NS300 (NanoSight, Amesbury, UK) and the NanoFCM Flow NanoAnalyser (NanoFCM, Xiamen, China) were used to analyze the size distribution and concentration of exosomal particles.

**Proteomics procedures**

The exosomes were lysed with 8 M urea (Sigma-Aldrich, Shanghai, China) and 1% Phenylmethylsulfonyl fluoride (Beyotime, Shanghai, China) and their protein contents were reduced and alkylated using dithiothreitol and iodoacetamide, respectively (Sigma-Aldrich). Next, Lys-C and trypsin (Beijing Life Proteomic, Beijing, China) were used to digest proteins into peptides, and the peptide samples were separated on a nanoElute Ultra High-Performance Liquid Chromatography (Bruker Daltonics, Bremen, Germany) fitted with a C18 column. The system was then coupled to a *timsTOF* Pro mass spectrometer operating in positive mode with enabled trapped ion mobility spectrometry (TIMS) and a 100% duty cycle (100 ms ramp time). The raw data were processed using Spectronaut v17 by matching to the human proteome (UniProt, November 2022), which contained 20,397 entries without isoforms. Differential analysis of the protein profile dataset was performed with a false discovery rate (FDR) threshold of 0.05. Pathway enrichment analysis was performed using the KEGG, and the Fisher exact test was used to determine the statistical significance of expression differences.

**Single-cell RNA Sequencing Data Analysis**

To elucidate the potential cellular sources of serum exosomal protein biomarkers, we analyzed single-cell RNA sequencing (scRNA-seq) datasets derived from patients with ILD. We specifically utilized the publicly available human lung scRNA-seq datasets published by Heini M Natri et al. (PMID: 38548990), accessible through the NCBI Gene Expression Omnibus (GEO) database (Accession number: GSE227136). The following software and tools were used: Seurat v5.1 for data processing and analysis and R v4.4.1 for scripting and statistical analyses.

**Enzyme-linked immunosorbent assay**

ELISA were performed as previously described^18^. Serum and exosomal levels of KL-6, CAPN2, and SP-B in both healthy controls (HC) and ILD patients were quantified using the following kits: human KL-6 kit (ELK biotechnology, Cat# ELK9353), human CAPN2 kit (Jianglai, Cat# JL53051), human SP-B kit (Jianglai, Cat# JL11070), respectively, according to the manufacturers’ instructions.

**Immunostaining**

Lung tissue samples from healthy donors, patients with ILD, and paracancerous tissue from lung cancer patients were obtained from the Biorepository and Precision Pathology Center (BRPC) at the First Affiliated Hospital of Guangzhou Medical University. The acquisition of these tissues was conducted under GYFYY Institutional Review Board (IRB)-approved protocol (#2022-88).

Formalin-fixed, paraffin-embedded human lung tissue sections (4-µm thick) were deparaffinized using xylene and rehydrated using an ethanol gradient (100%, 95%, 70%, 0%). Immunohistochemistry of tissue sections was performed after inactivation of endogenous peroxidase with H_2_O_2_ in methanol. To enhance antigen retrieval, sections were kept in citrate buffer (0.01 M, pH 6.0) for 10 min and then microwaved. After blocking the sections with 5% donkey serum in 0.2% Triton-X100/PBS, the primary antibodies were applied overnight at 4 °C. After washing, the sections were incubated with HRP-conjugated secondary antibodies, counterstained with hematoxylin, and finally imaged on a Leica Aperio Versa 8 microscope. For immunofluorescence, the sections were incubated with fluorescently conjugated secondary antibodies for 1h at room temperature, in the dark. The nuclei were counterstained with DAPI (1:5000, Beyotime, Cat#P0131) for 5 min. Confocal images were obtained using the ZEISS LSM 800 confocal microscope and processed using Zeiss ZEN 3.8 software. The following primary antibodies were used: mouse anti-MUC1 (1:500, Santa Cruz, Cat# sc-7313), mouse anti-CAPN2 (1:500, Proteintech, Cat# 66977-1-Ig), rabbit anti-SP-B (1:500, FineTest, Cat# FNab07797), anti-SP-C (1:1000, Sigma-Aldrich, Cat# AB3786). The following secondary antibodies were used: goat anti-mouse IgG HRP (1:1000, Invitrogen, Cat# G-21040) and goat anti-rabbit IgG HRP (1:1000, Invitrogen, Cat# G-21234), goat anti-rabbit Alexa Fluor 488 (1:2000, Abcam, Cat# Ab150077) or goat anti-mouse Alexa Fluor 555 (1:2000, Abcam, Cat# Ab15014).

**Western blotting**

Serum samples and exosomal lysates were treated with RIPA lysis buffer (Beyotime). After separation of lysates by SDS-PAGE, the proteins were transferred onto polyvinylidene fluoride membranes. The membranes were blocked for 1 h at room temperature, and then incubated with primary antibodies overnight at 4 ℃. After washing and incubation with secondary antibodies, the blots were developed using the enhanced chemiluminescence (ECL) reagent (Servicebio, Wuhan, China) and imaged using the Tanon-5200 chemiluminescence imaging system (Tanon, Shanghai, China). The following primary antibodies were used: mouse anti-MUC1 (1:2000, SantaCruz, Cat# sc-7313), mouse anti-CAPN2 (1:3000, Proteintech, Cat# 66977-1-Ig), mouse anti-SP-B (1:1000, Santa Cruz, Cat# sc-13314), rabbit anti-SP-D (1:3000, Abcam, Cat# ab220423). The following secondary antibodies were used: goat anti-mouse IgG HRP (1:3000, CST, Cat# 7074S) and goat anti-rabbit IgG HRP (1:3000, CST, Cat# 7076S).

**Nanogold labeling of exosomal proteins**

The mouse anti-MUC1 (Santa Cruz, Cat# sc-7313), mouse anti-CAPN2 (Proteintech, Cat# 66977-1-Ig), and mouse anti-SP-B (Santa Cruz, Cat# sc-13314) antibodies were biotinylated using Biotin-NHS (Thermo, Shanghai, China) according to the manufacturer's instructions. Exosomal samples were then incubated with the biotinylated antibodies overnight at 4 ℃, combined with streptavidin-labeled 10-nm colloidal gold particles (kindly provided by J. Huiyang, PMID:38621340), and examined by TEM (FEI, Tecnai G2 Spirit).

**Chemiluminescent immunoassay**

Magnetic beads were prepared by sequentially exposing the magnetic bead solution (10 mg/mL) to the EDC solution and then the NHS solution. The supernatant was removed, and the resuspended beads were incubated with 200 μg of capture antibody at room temperature for 2 h. Next, the beads were incubated with 100 μL of blocking agent at RT for 1 h, after which the supernatant was removed. The bead suspension was sonicated on a touch-sensitive ultrasonic cell disruptor at 10% power, and the supernatant was discarded. Finally, the coated magnetic beads were diluted with diluent buffer (50 mM MES buffer with 0.1% TRIS, 0.9% NaCl, 3% BSA, 0.1% Tween-20, and 0.1% sodium azide) to obtain a 0.8 mg/mL working solution. For detection antibody labeling, 200 μg of detection antibody and 25 μL of NHS-coupled acridine ester (10 mg/mL) were incubated with 500 μL of the pre-prepared labeling buffer for 60 min. The reaction was then quenched with 100 μL of 10% lysine at room temperature for 30 min. Unreacted substances were removed on a desalting column, and the resulting magnetic beads labeled with the detection antibody (the detection reagent) were diluted with a labeling preservation solution (PBS containing 0.2% BSA and 0.1% PC300) to a concentration of 200 μg/mL. The solution was then further diluted with diluent buffer (50 mM MES containing 0.9% NaCl, 2% BSA, 0.1% Tween-20, and 0.1% Proclin 300) to a 0.4 μg/mL working solution. The detection reagent was subsequently mixed with the diluted exosomal extract and analyzed on the Shine i2000 Series Fully Automated CLIA Analyzer (Fapon Biotech, Guangdong, China). The following primary antibodies were used: Capture antibody of KL-6 (MBL Beijing Biotech, Cat# 8MKL-61); Conjugate antibody of KL-6 (MBL Beijing Biotech, Cat# 8MKL-62); Capture antibody of CAPN2 (in house); Conjugate antibody of CAPN2 (in house); Capture antibody of SP-B (in house); Conjugate antibody of SP-B (in house); The chemiluminescence values of exosomal KL-6 were measured using the Lumipulse G KL-6 detection kit (Fujirebio, Tokyo, Japan) and normalized to the KL-6 protein concentration. The chemiluminescence standard curves for other exosomal proteins, including CAPN2 (BBI, Cat# D620329) and SP-B (FEIYUE, Cat# FY-P517069), were generated using commercially available antigens.

**Key resources table**

| REAGENT or RESOURCE | SOURCE | IDENTIFIER |
| --- | --- | --- |
| Antibodies | | |
| mouse anti-MUC1 | Santa Cruz | Cat# sc-7313 |
| mouse anti-CAPN2 | Proteintech | Cat# 66977-1-Ig |
| rabbit anti-SP-B | FineTest | Cat# FNab07797 |
| anti-SP-C | Sigma-Aldrich | Cat# AB3786 |
| goat anti-mouse IgG HRP | Invitrogen | Cat# G-21040 |
| goat anti-rabbit IgG HRP | Invitrogen | Cat# G-21234 |
| goat anti-rabbit Alexa Fluor 488 | Abcam | Cat# Ab150077 |
| goat anti-mouse Alexa Fluor 555 | Abcam | Cat# Ab15014 |
| rabbit anti-SP-D | Abcam | Cat# ab220423 |
| Capture antibody of KL-6 | MBL Beijing Biotech | 8MKL-61 |
| Conjugate antibody of KL-6 | MBL Beijing Biotech | 8MKL-62 |
| Capture antibody of CAPN2 | This paper | NA |
| Conjugate antibody of CAPN2 | This paper | NA |
| Capture antibody of SP-B | This paper | NA |
| Conjugate antibody of SP-B | This paper | NA |
|  |  |  |
| Biological samples |  |  |
| human serum samples | the First Affiliated Hospital of Guangzhou Medical University (GYFYY) | Healthy adult donors and ILD patients. |
| human serum samples | Shantou Central Hospital (STC) | Healthy adult donors and ILD patients. |
| human serum samples | the First Affiliated Hospital of Chongqing Medical and Pharmaceutical College (CQMPC) | Healthy adult donors and ILD patients. |
| Human lung tissues. | Biorepository and Precision Pathology Center (GYFYY) | Recipients and donors in lung transplantation surgery. |
|  |  |  |
| Chemicals, peptides, and recombinant proteins | | |
| CAPN2 antigen | BBI | Cat# D620329 |
| SP-B antigen | FEIYUE | Cat# FY-P517069 |
| Endopeptidase Lys-C | Beijing Life Proteomic | Cat# HLS LYS001C |
| trypsin | Beijing Life Proteomic | Cat# HLS TRY001C |
|  |  |  |
| Deposited data | | |
| Raw mass spectrometry data and Spectronaut output tables | This paper | NA |
| Single-cell RNA Sequencing Data | Natri HM et al. *Nat Genet* 2024 Apr;56(4):595-604. PMID: [38548990](https://www.ncbi.nlm.nih.gov/pubmed/38548990) | https://www.ncbi.nlm.nih.gov/geo/query/acc.cgi?acc=GSE227136 |
|  |  |  |
| Software and algorithms | | |
| R using the EDGER (version 4.2.1), Seurat (version 5.1), Tableone (version 0.13.2) | NA | NA |
| GraphPad Prism (version 8.02) | NA | NA |
| SPSS Statistics version 26.0 | IBM | NA |
| G-Power 3.1 | NA | NA |
|  |  |  |
